# Engineered carbon-based fertilizers for sustainable agriculture: physicochemical mechanisms of nutrient release, soil health improvement, and plant uptake

**DOI:** 10.1039/d6ra05512e

**Published:** 2026-10-09

**Authors:** Othman Al-Dossary, Tauqeer Ahmed Qadri, Rashid Abbas Khan, Abdul Waheed, Bader Alsubaie, Wael F. Shehata, Jameel M. Al-Khayri, Amjid Khan

**Affiliations:** a Department of Agricultural Biotechnology, College of Agriculture and Food Sciences, King Faisal University Al-Ahsa 31982 Saudi Arabia othmand@kfu.edu.sa subaiebys@kfu.edu.sa wshehata@kfu.edu.sa jkhayri@kfu.edu.sa; b UNESCO-UNISA Africa Chair in Nanoscience and Nanotechnology, College of Graduate Studies, University of South Africa 1 Preller Street, Muckleneuk, Ridge, P.O. Box 392 Pretoria Gauteng Province 0003 South Africa rashidabbaskhan98@gmail.com mrtauqeer0027@gmail.com khana2@unisa.ac.za laghari_awl@yahoo.com

## Abstract

The intensive use of synthetic mineral fertilizers has sustained global crop productivity but has also contributed to greenhouse gas emissions, nutrient losses, and progressive soil degradation. Carbon-based fertilizers (CBFs) represent an emerging class of functional carbon materials that integrate nutrient retention and delivery with carbon stabilization and soil–rhizosphere interactions. CBFs can be derived from diverse biomass feedstocks, including crop residues, livestock manure, and organic waste, through thermochemical and biological conversion routes such as pyrolysis, hydrothermal carbonization, composting, and anaerobic digestion. This review critically examines the evolution of CBFs from conventional carbon-rich soil amendments toward engineered nutrient-delivery systems with tailored physicochemical and interfacial properties. Particular emphasis is placed on carbonization and activation strategies; pore architecture; specific surface area; surface charge; oxygen-containing functional groups; and surface functionalization, and on their influence on nutrient adsorption–desorption, ion exchange, complexation, diffusion, and controlled release. Advanced modification strategies, including chemical activation, co-pyrolysis, mineral–organic functionalization, and nano-carbon integration, are further evaluated, with emphasis on nano-biochar, graphene derivatives, and carbon dots. The review also examines interactions among carbon matrices, nutrients, and rhizosphere microorganisms, distinguishing established physicochemical and microbial mechanisms from emerging stimuli-responsive and nano-enabled delivery concepts. Finally, the laboratory-to-field translation gap is critically assessed, highlighting how soil heterogeneity, environmental variability, material transformation, and microbial dynamics influence CBF performance and identifying priorities for reproducible, field-validated, and environmentally responsible fertilizer development.

## Abstract 2

Biomass feedstocks are converted into engineered carbon-based fertilizers whose pores and surface chemistry control nutrient release, rhizosphere interactions, and plant uptake, improving soil health and sustainability.

## Graphical abstract

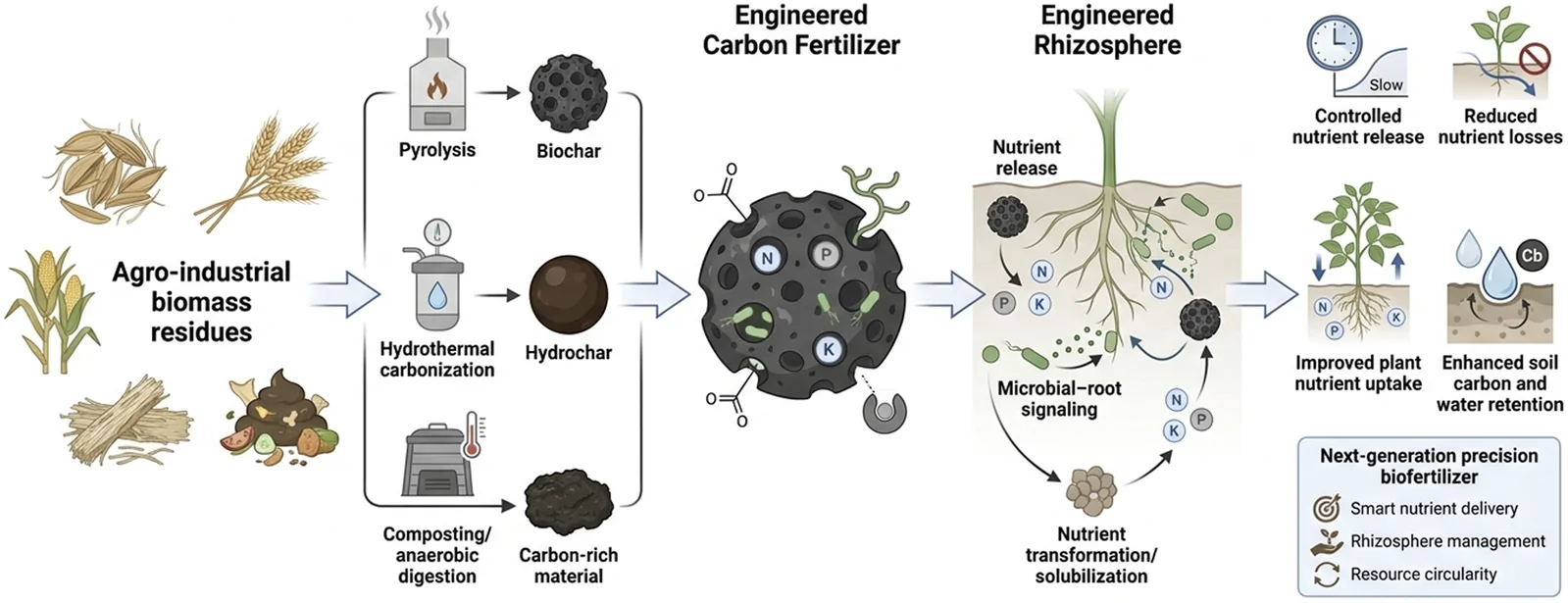

## 1. Introduction

The intensification of modern agriculture since the green revolution has relied primarily on synthetic fertilizers to maintain global crop productivity and food security. Nitrogen (N), phosphorus (P), and potassium (K) fertilizers have led to significant increases in agricultural production and currently account for a large fraction of food production in the world.^1^ However, the vast use of synthetic fertilizers has caused serious environmental and sustainability concerns. The nitrogen fertilizer production, which in most cases uses the energy-intensive Haber–Bosch process, needs large amounts of fossil fuel and produces significant amounts of greenhouse gas (GHG) emissions.^2^ Estimates show that the global supply chain of synthetic nitrogen fertilizers accounts for over 1.1 Gt CO_2_-eq in emissions every year, representing a significant share of the agricultural food system's emissions. Furthermore, the production of nitrogen fertilizer alone is responsible for about 0.41 Gt carbon dioxide equivalent per annum, which is a measurable part of total global greenhouse gas emissions.^3^

Beyond the carbon costs of production, the application of fertilizer in agricultural soils also contributes to environmental degradation through multiple pathways. Microbial transformations of target nitrogen often lead to nitrous oxide (N_2_O) emissions, which is a climate-warming compound having a global warming potential nearly 300 times greater than carbon dioxide.^4^ In addition, inefficient nutrient utilization causes nitrate to be leached, volatilized, and run off instead, and so enter groundwater and contribute to eutrophication of aquatic ecosystems.^5^ Studies estimate that the use of synthetic nitrogen fertilizer significantly increases farm-level greenhouse gas emissions and is a major source of nutrient pollution in our agroecosystems. Such environmental impacts were highlighted, which led to the rise of the need for alternative fertilization strategies that improve nutrient efficiency while decreasing the ecological footprint of crop production.^6^

Agriculture produces large amounts of organic residues, such as crop residues, animal manure, and food waste, as well as other biomass streams. Much of this biomass is under-exploited or mismanaged, causing further environmental costs in the form of methane and nutrient emissions and waste disposal issues.^7^ In recent years, the concept of circular bioeconomy has emerged as a promising concept to counter these challenges by transforming waste streams and organic neutral processes into locally and regionally derived resource inputs for farmland.^8^ Bio-based fertilizers made from biomass have the potential to recover nutrients and organic carbon while reducing environmental impact compared to conventional fertilizers. Recent analyses have shown that nutrient recovery technologies such as composting, anaerobic digestion, and biochar production enable 60 to 95% nutrient recovery efficiency from organic waste streams along with improved soil physical and biological properties.^9^

Carbon-based fertilizers (CBFs) have attracted increasing interest as a new generation of sustainable fertilization technologies. Carbon-based fertilizers are normally produced from organic biomass by means of various processes such as pyrolysis, composting, hydrothermal carbonization, or bio-digestion, generating carbon-rich materials with the ability to interact with soil nutrients and soil microbial communities.^10^ Biochar-based fertilizers, carbon materials derived from compost, and emerging carbon nanomaterials are important examples of this class of fertilizers. These materials have unique physicochemical properties such as high surface area, porous structure, and functionalized carbon surfaces, allowing them to adsorb nutrients, control their release, and to promote microbial activity in the rhizosphere.^11^ Consequently, the application of carbon-based fertilizers has the benefit of recycling organic waste but also adds to carbon sequestration in soil, better nutrient retention, and soil health.^10^

There has now been substantial evolution in the understanding of carbon-based materials in agricultural science. Early research has been mainly focused on biochar as a soil amendment to improve the soil structure and fertility.^12^ However, there has been an emphasis on the potential of developed carbon materials as active nutrient carriers and delivery platforms. Through chemical functionalization, nutrient impregnation, and nanoengineering approaches, carbon matrices can be developed into novel types of fertilizers that offer the potential for the controlled release of nutrients as well as targeted interaction with plant-microbe systems.^13^ This conceptual evolution represents a wider shift in science around fertilization – from reactionary and slow-moving treatment of carbon materials as amending and passive elements of the soil, to accepting them as fertilizers with functions as integrated fertilizers and complex functions that include carbon sequestration, nutrient management, and rhizosphere engineering.^14^

Unlike previous reviews largely focused on biochar-based slow/controlled-release fertilizers and their formulation, nutrient-release behavior, and agronomic effects, this review establishes a broader carbon-based fertilizer framework encompassing biochar, biomass-derived carbon materials, carbon–microbial composites, and emerging carbon nanomaterials. It integrates feedstock and conversion technologies, carbon–nutrient interactions, nutrient-release mechanisms, rhizosphere engineering, plant responses, carbon sequestration, circular bioeconomy, and environmental and regulatory considerations. This perspective highlights the transition from passive carbon amendments to multifunctional carbon fertilizer platforms for precision and low-carbon agriculture.

## 2. Evolution and classification of carbon-based fertilizers (CBFs)

The growing need for sustainable nutrient management has led to rapid interest in materials derived from carbon extraction as alternative fertilizer-supply systems. In recent years, CBFs have become an important group of soil inputs that can simultaneously increase nutrient use efficiency, soil quality, and carbon sequestration.^15^ Unlike traditional mineral fertilizers, which primarily supply nutrient elements in readily soluble forms, CBFs combine organic carbon matrices with nutrient-delivery functions, thereby enabling more controlled interactions within the soil–plant–microbes continuum. The conceptual framework of CBFs has developed very rapidly from the simple organic amendments used to engineered nutrient platforms to optimize soil fertility whilst minimizing environmental impacts.^10^

### 2.1. Characteristics of CBFs

Carbon-based fertilizers are generally understood as fertilizers derived from organic biomass, in which organic matrices containing carbon serve as carriers, regulators, or enhancers of plant nutrients. These fertilizers are generally produced through biological, thermochemical, or physicochemical processes, including composting, pyrolysis, hydrothermal carbonization, and anaerobic digestion.^10^ The resulting materials have stable organic carbon structures and can interact with nutrients, soil minerals, and the microbial community. CBFs are carbon frameworks, often aromatic, arranged into porous architectures and bearing surface functional groups, including hydroxyl, carboxyl, and carbonyl moieties.^16^ These structural characteristics have several functional advantages, such as high surface area, high adsorption capacity, and high capacity to hold water and nutrients in soil systems. As a result, carbon matrices can act as nutrient reservoirs, reducing nutrient losses (leaching and volatilization) and favoring gradual nutrient release to plants.^17^

Another important feature of CBFs is their ability to regulate the biological processes in the rhizosphere. The porous nature of carbon materials creates microhabitats, which support the colonization and formation of microbial biofilms for enhanced microbial-mediated nutrient transformation for N fixation, mobilization of P, and decomposition of organic matter.^18^ In addition, the redox-active properties of some carbon materials can lead to electron-transfer processes that affect biochemical reactions in the soil and nutrient cycling. Collectively, these physicochemical and biological attributes lead to the differences between carbon-based fertilizers and conventional fertilizers due to their ability to accomplish integrated nutrient delivery, soil carbon stabilization, and rhizosphere regulation in a single system.^19^

### 2.2. Functional classification of carbon-based fertilizers

Carbon materials are distinguished according to their primary intended function. Unmodified biochar, compost-derived carbon, and related materials applied primarily to improve soil structure, water retention, carbon storage, or microbial habitat are classified as carbon-based soil amendments.^20^ In contrast, engineered carbon-based fertilizers are carbon matrices intentionally functionalized, enriched, or combined with nutrients and/or beneficial microorganisms to provide nutrient delivery, controlled release, or microbial carrier functions.^10^ Accordingly, nutrient-enriched carbon materials are classified as CBFs when nutrient delivery is an explicit design objective. In contrast, carbon materials may function as microbial carriers when beneficial microorganisms are deliberately immobilized or delivered through the carbon matrix. These functions may coexist in multifunctional CBFs, but classification is based on the primary intended function.^21^

Biochar-based fertilizers are among the most well-developed types of CBFs. Biochar is obtained by the pyrolysis of organic biomass under limited-oxygen conditions, and the resulting carbonaceous material is stable, highly porous, and exhibits strong adsorption capacity. When enriched with nutrients through impregnation, co-composting, or mineral blending, biochar has the function of a slow-release fertilizer that enhances the quality of nutrient retention and soil fertility.^11^

Compost-derived carbon fertilizers are derived from the aerobic decomposition of organic wastes, including crop residues, manure, and food waste. Composting is the method that turns unstable organic matter into humified carbon compounds that are important nutrients as well as soil structure.^22^ Carbon-rich compost products may be further adjusted or combined with mineral nutrients to enhance their fertilization efficiency. Digestate and manure carbon fertilizers are produced by anaerobic digestion of organic waste for biogas production. The undigested digestate is a partially stabilized organic carbon with valuable quantities of nitrogen, phosphorus, and micronutrients.^23^ When processed or combined with carbon materials like biochar, digestate-derived fertilizers possess a high potential to be used as valuable nutrient sources together with lowered nutrient losses.^24^

A further evolving form is that of carbon-microbial biofertilizers, in which the carbon form is combined with stronger and beneficial microorganisms.^25^ In these systems, carbon materials such as biochar or compost serve as a microbial carrier for the survival and activity of plant growth-promoting microorganisms, including nitrogen-fixing, phosphate-solubilizing, and other rhizosphere-beneficial organisms. Such combinations help enhance microbial colonization with nutrients and their transformation processes in the soil.^26^

More recently, advances in nanotechnology have led to the development of carbon nanofertilizers, which are nanostructured carbon materials, including nano-biochar, carbon nanotubes, graphene derivatives, and carbon dots. Such materials possess distinctive physicochemical features, including high surface reactivity and nanoscale porosity, which enable precise nutrient loading and control of nutrient delivery. Although still in an early stage of development, carbon nanofertilizers offer a promising frontier for precision agriculture and smart fertilization technology.^27^

### 2.3. Evolution from traditional biochar to smart carbon platforms

The science of carbon-based materials in agriculture has changed incrementally over the past two decades. Early research focused largely on the application of biochar as a soil amendment, inspired by the fertile “terra preta” soils of the amazon basin.^28^ In this context, the main attributes of biochar were considered to be improvements in soil physical properties, water retention, and organic carbon content of soils.^29^ Subsequent research indicated that carbon materials could also react with nutrients through adsorption, ion exchange, and surface complexation.^30^ This realization led to the development of biochar-based fertilizers, in which nutrient-enriched carbon matrices were created to hold nitrogen, phosphorus, potassium, or micronutrients, thereby promoting nutrient retention and reducing nutrient losses.^11^

In recent years, studies have become focused on the engineering of carbon materials with specific physicochemical properties that can provide it with the role of active components of nutrient management systems.^31^ One of the biggest trends is carbon surface functionalization, in which chemical alteration introduces functional groups that increase nutrient-binding capacity, catalytic activity, or microbial interactions. Such modifications may include oxidation, mineral coating or addition of metal ions for improved nutrient loading and nutrient release characteristics.^32^ Another key development is the development of approaches with nutrient engineering intention, in order to design carbon matrices that can adjust the kinetics of nutrient release according to the environmental conditions.^33^ Through techniques such as nano-encapsulation, mineral impregnation, and hybrid composite formation, it is possible to tailor carbon-based fertilizers to achieve gradual, efficient nutrient release, thereby increasing nutrient use efficiency while minimizing environmental losses. These technological advances have led to the concept of stimuli-responsive carbon-based fertilizers, *i.e.*, carbon materials that are chronically combined with advanced delivery systems, microbial interactions and environmental responsiveness.^34^

In such systems, these carbon matrices are not only passive carriers of carbon components but also serve as multifunctional platforms that can sense general soil conditions and nutrient availability, and enhance beneficial processes in the rhizosphere.^35^ This conceptual evolution represents a shift from the traditional use of soil amendments to intelligent fertilizer systems that enhance sustainable and climate-resilient agriculture.^36^ The development of carbon-based fertilizers represents a broader wave in agricultural innovation, in which the fields of materials science, biotechnology, and environmental engineering converge to develop next-generation nutrient management strategies. Understanding this evolution is key to developing carbon-based fertilizer systems that deliver the greatest agronomic benefit while supporting global efforts for low-carbon, resource-efficient agriculture.^37^Fig. 1 establishes the conceptual foundation of carbon-based fertilizers by illustrating their progression from conventional organic amendments and unmodified biochar toward increasingly engineered nutrient-delivery platforms. The upper progression emphasizes how physical, chemical, and biological modification can alter carbon pore architecture and oxygen-containing surface functionalities, including carboxyl, hydroxyl, and carbonyl groups, thereby creating sites for nutrient retention and exchange. The figure further illustrates the dual functionality of the carbon matrix as an abiotic nutrient reservoir and a porous habitat for beneficial microbial biofilms. The lower classification framework organizes carbon-based fertilizer technologies into distinct functional categories, linking increasing material complexity with enhanced opportunities for nutrient management and rhizosphere engineering.

**Fig. 1:**
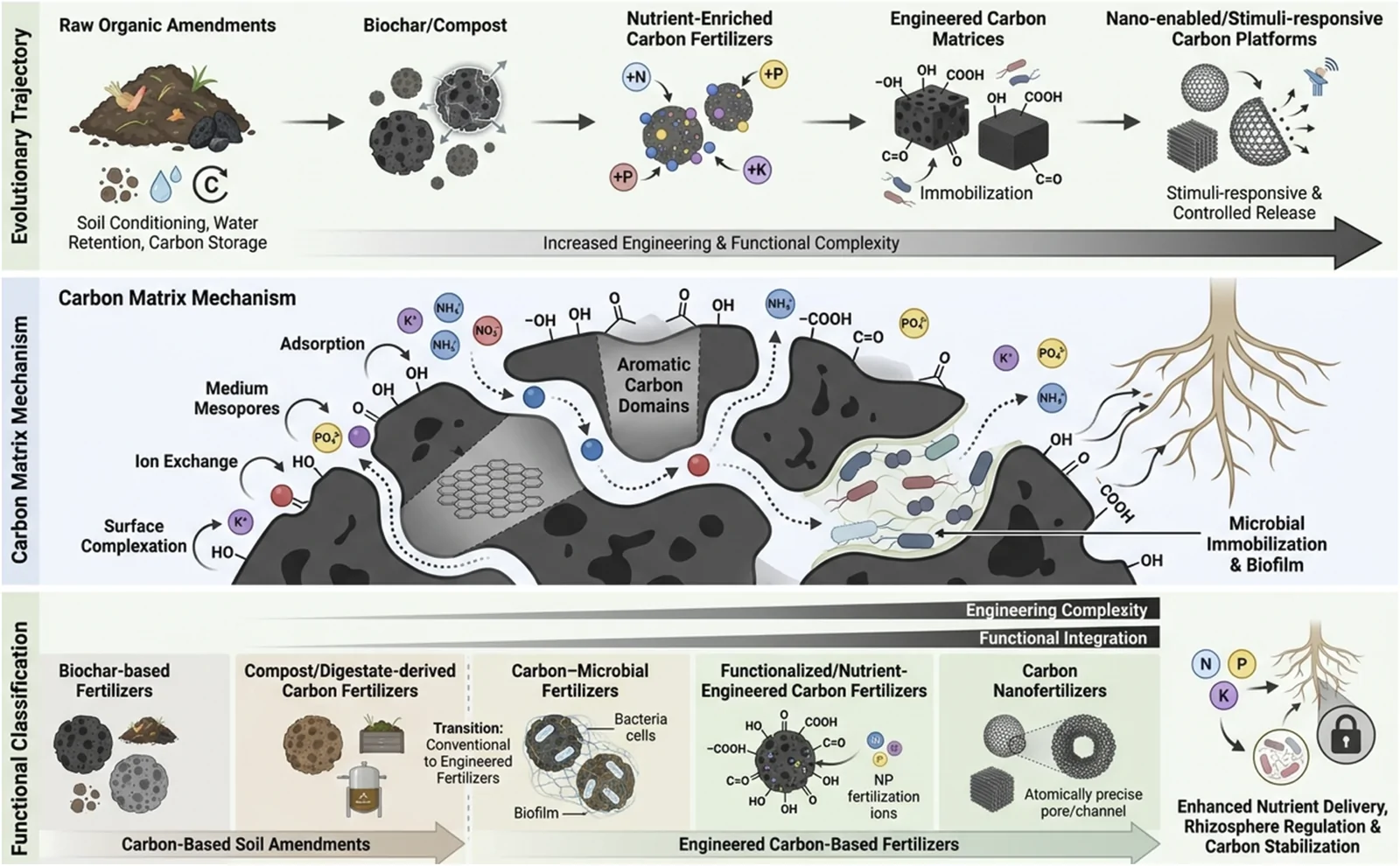
Evolution, classification, and functional transition of carbon-based fertilizers. The figure illustrates the progression from conventional carbon-rich amendments toward engineered nutrient-delivery platforms and nano-enabled carbon systems. Increasing material engineering introduces controlled nutrient retention and release, surface functionalization, microbial carrier functions, and emerging condition-responsive delivery concepts (Fig. 1 partly generated by Google Gemini and figure Labs).

## 3. Biomass feedstocks and carbonization technologies

The performance and agronomic functionality of CBFs depend heavily on the biomass feedstock and the technologies used in their production. Biomass resources exhibit large variation in chemical composition, including carbon content, lignocellulosic structure, nutrient concentrations, and mineral fractions.^23^ Consequently, the transformation of these feedstocks through thermochemical or biological processes determines the structural properties, nutrient retention capacity, and stability of the resulting carbon materials. A thorough understanding of the relationship among biomass origin, carbonization routes, and fertilizer functionality is therefore essential for the rational design of CBFs.^38^

### 3.1. Biomass source of CBF production

A wide array of organic biomass resources can be used as raw materials in the production of carbon-based fertilizers. These feedstocks vary in nutrient content, lignin and cellulose content, and mineral phases, which affect the physicochemical properties of the carbon materials produced from them.^39^ Agricultural residues are among the most plentiful and readily available sources of biomass for producing CBF. Crop residues, including rice husks, wheat straw, corn stover, sugarcane bagasse, and other plant by-products, are produced in large quantities all over the world. These materials have high levels of lignocellulosic carbon and moderate content of nutrients such as potassium, phosphorus, and micronutrients.^40^ When used as biochar or compost (derived fertilizer), agricultural residues will serve as stable carbon matrices with the potential to transform soils from deteriorating to sound, improving water retention and nutrient adsorption. Utilizing these residues for production of fertilizers also plays a role in the decrease of open field burning and the associated air pollution.^41^

Another important source of biomass is livestock manure, including poultry, cattle, and swine. Animal manures generally have greater quantities of nitrogen, phosphorus, and organic matter than do plant residues.^42^ When recycled through pyrolysis or composting processes, manure-derived carbon materials have the potential to serve as nutrient-rich fertilizers and at the same time to reduce odor, pathogen loads, and remote environmental contamination resulting from the raw application of manure.^43^ Food waste as well as municipal organic waste are even becoming more recognized as a resource to be used in nutrient recycling in circular bioeconomy systems.^44^ Urbanization and food processing industries produce huge amounts of organic waste streams that have to be disposed of and landfilled and are a major source of emissions of methane and pollutants to the environment.^45^ Converting these wastes into carbon-based fertilizers through composting, hydrothermal carbonization, or anaerobic digestion processes allows the organic carbon and other essential nutrients to be recovered, and thus waste management issues become an agricultural input.^46^

Apart from agricultural and municipal sources, industrial organic biomass such as paper mill sludge, brewery waste, distillery residues and other agro – industrial by – products can also be used for CBF production.^47^ These materials often contain large amounts of organic carbon and mineral nutrients, which can stabilize carbonization processes. The valorization of industrial organic residue into the industrial production of fertilizers can contribute to the valorization of waste and minimize the environmental burden from industrial waste disposal.^48^

### 3.2. Carbon conversion technologies

The conversion of biomass to functional carbon-based fertilizers using various conversion technologies such as biological and thermochemical. These processes modify the chemical composition and structural properties of the raw biomass to yield carbons with unique physical and chemical properties.^49^ Pyrolysis is one of the most commonly used technology. This is a thermochemical process in which biomass is heated in the presence of limited oxygen, at temperatures that are usually 300 to 700 °C.^50^ Pyrolysis is a process of breaking down the organic matter to produce biochar, bio-oil and syngas with biochar being the condensed carbon-rich solid fraction of the process. Biochar derived using pyrolysis technologies has an almost porous structure, is composed of aromatic carbon networks and has a high surface area, making it suitable for nutrient adsorption and soil amendment applications. By adjusting the pyrolysis conditions or including nutrient sources during this production process, improper biochar can be developed into fertilizer with biochar-based nutrient release properties.^51^

Another important technology is hydrothermal carbonization (HTC). HTC is used to convert wet biomass to produce carbon-rich hydrochar under high pressure and moderately high temperatures (normally 180–250 °C) in aqueous conditions. Unlike pyrolysis, HTC can handle biomass with a high moisture content without the absence of the drying process, so it is especially suitable for food waste and sewage sludge and other kinds of wet organic waste.^52^ Hydrochar often contains increased amounts of oxygen high functional groups and nutrients than biochar and may improve the interaction of nutrients and microbial communities with soil.^53^

Composting is a biological conversion process involving the use of microorganisms to aerobically break down organic materials to produce stabilized organic matter that is rich in humic substances. Compost-derived carbon fertilizers help increase soil fertility by providing nutrients and enhancing soil structure, microbial and water retention properties.^54^ When combined with carbon rich materials (*e.g.* biochar), the nutrient stabilization along with microbial colonization can be further maximized by the composting process.^55^

Anaerobic digestion technology is a widely utilized technology producing biogas from organic wastes by microbial decomposition under oxygen absence. Partially stabilized organic carbon and large amounts of plant nutrients are found as a residual by-product known as digestate.^56^ Digestate can be used directly as a fertilizer or further processed to produce improved, nutrient stable fertilizers with reduced environmental risks by being enriched carbon. Recent developments in biomass processing have also resulted in integrated hybrid processes, in which a combination of conversion technologies is used in order to increase the functionality of carbon-based fertilizers.^57^ For example, biochar can be co-composted with organic waste so that nutrient-enriched biochar-compost fertilizers can be produced, while digestate can be further carbonized to produce stabilized carbon materials with better agronomic properties. Such hybrid approaches are facilitating integrating nutrient recovery, carbon stabilization and waste management in the one production system.^58^

### 3.3. Process parameters governing carbon properties

The physicochemical features of the carbon-based fertilizers are greatly affected with the process parameters for the transformation of biomass, and this affects the structure, stability, and nutrient retention capacity of the resulted carbon materials.^15^ Temperature is a very important factor that determines the level of carbonization and the structure of biochar or hydrochar. Higher temperatures of pyrolysis usually enhance the formation of aromatic carbon, surface area, and of structural stability and decrease volatile compounds. However, excessively high temperatures may result in reduced abundance of oxygen-containing functional groups that are of importance for nutrient adsorption and cation exchange processes.^59^ Residence time or the extent of time that biomass is exposed to carbonization conditions also influences the chemical composition and porosity of carbon materials. Longer residence times usually encourage increased stabilization of carbon and increased structural development but as processing time is long nutrient content risks being decreased because of thermal decomposition or volatilization.^60^

Another important factor is the limitation of oxygen, which determines which type of carbonization pathway is going to take place. Thermochemical processes like pyrolysis need a low oxygen condition so that combustion and carbon structure destruction is prevented. Differences in the availability of oxygen affect the development of pores and aromatic carbon structures and the type of minerals that form in the resulting material.^61^ Activation treatments are often used to increase the functionality of carbon-based fertilizers. Physical activation methods such as steam or carbon dioxide treatment process increase the porosity and surface area while the chemical activation with acids, bases or mineral salts introduce new functional groups improving the adsorption and retention of nutrients.^62^ These activation strategies allow for the tailored use of carbon matrices in a specific application for fertilizer applications, including the loading of nutrients, the adsorption of pollutants and microbial colonization.^32^ The relationship between biomass origin, conversion technology and functional fertilizer properties is summarized in Table 1.

**Table 1:** Biomass feedstocks, conversion technologies, and functional properties of carbon-based fertilizers

Feedstock	Conversion technology	Key physicochemical traits	Functional properties/agronomic benefits	References
Rice husk, wheat straw, corn stover	Pyrolysis (300–700 °C)	High aromatic C, porous structure, high surface area, cmolc kg^−1^ 20–60 cmolc kg^−1^	Slow nutrient release, enhanced water retention, improved soil structure, pollutant adsorption	63
Poultry litter, cattle/swine manure	Composting/co-pyrolysis	Humified organic matter, moderate nutrient content (N,P,K), moderate CEC	Nutrient enrichment, microbial habitat formation, soil fertility improvement	64
Food waste, municipal organic waste	Hydrothermal carbonization (180–250 °C)	Oxygen-rich functional groups, high water retention capacity, moderate porosity	Nutrient recovery, improved microbial activity, reduced environmental pollution	65
Industrial residues (brewery, paper sludge, agro-industrial by-products)	Anaerobic digestion/hybrid carbonization	Partially stabilized organic carbon, high N & P content, mineral retention	Enhanced nutrient availability, bioenergy co-production, circular economy integration	66
Biochar–microbial composites	Pyrolysis + microbial inoculation	Porous carbon matrix with microbial immobilization sites	Supports PGPR, enhances nutrient transformation, improved plant growth & stress tolerance	67
Carbon nanomaterials (graphene, carbon dots, nano-biochar)	Nano-engineering/functionalization	High surface reactivity, nanoscale porosity, tunable functional groups	Precision nutrient delivery, controlled release, targeted rhizosphere modulation	68

## 4. Physiochemical engineering of carbon matrices for fertilizer design

Carbon structural characteristics determine the physicochemical properties that ultimately govern CBF performance. Pore architecture, specific surface area, aromatic carbon domains, and oxygen-containing functional groups collectively determine the available reactive interfaces of carbon matrices. These structural attributes influence surface charge, cation exchange capacity, adsorption capacity, water retention, and the binding and release kinetics of nutrient ions.^69^ Consequently, differences in carbon architecture can regulate nutrient retention and availability, thereby affecting nutrient-use efficiency and reducing losses through leaching or volatilization. At the soil–plant interface, the porous structure additionally provides microsites for microbial attachment and activity, while reactive surface groups and redox-active sites can influence nutrient transformation and rhizosphere processes.^70^ Thus, the agricultural functionality of CBFs should be viewed as a sequential structure–property–function relationship, in which carbon architecture controls physicochemical behavior, which in turn determines nutrient-delivery efficiency, microbial interactions, and broader soil and plant responses.

### 4.1. Structural characteristics governing carbon-matrix functionality

Carbon-matrix performance is fundamentally determined by its structural architecture, including pore structure, specific surface area, surface chemistry, charge characteristics, and carbon stability. Pore size distribution and interconnected porosity determine the accessible surface area and provide physical domains for nutrient retention and microbial attachment.^71^ Higher surface area generally increases the number of available interaction sites, although its functional contribution depends on pore accessibility and surface chemistry. Surface functional groups, particularly hydroxyl (–OH), carboxyl (–COOH), and carbonyl (>C=O) groups, provide reactive sites that influence surface charge, ion exchange, and interactions with nutrient species.^72^ Surface charge further regulates the affinity of carbon matrices for ionic nutrients, whereas carbon stability determines the matrix's persistence and its contribution to long-term carbon storage. These structural attributes are strongly influenced by feedstock characteristics, carbonization conditions, and post-treatment processes, resulting in substantial variation among CBF materials.^73^ Collectively, the structural architecture establishes the physicochemical foundation that governs subsequent nutrient retention, release behavior, and biological interactions; these functional consequences are discussed in the following sections. Fig. 2 illustrates the structure–function relationship within engineered carbon matrices, showing how hierarchical pore networks ranging from micropores to mesopores and macropores determine the movement and retention of nutrients and water. The Fig. 2 connects these physical characteristics with surface-mediated processes such as adsorption, ion exchange, surface complexation, and reversible nutrient binding, while also showing how interconnected pores can provide pathways for diffusion and protected microsites for microbial colonization. These material-level properties are subsequently linked with agronomic functions, including sustained nutrient availability, improved water retention, reduced nutrient mobility and leaching, and enhanced microbial habitat formation, thereby demonstrating how carbon architecture can influence soil nutrient dynamics.

**Fig. 2:**
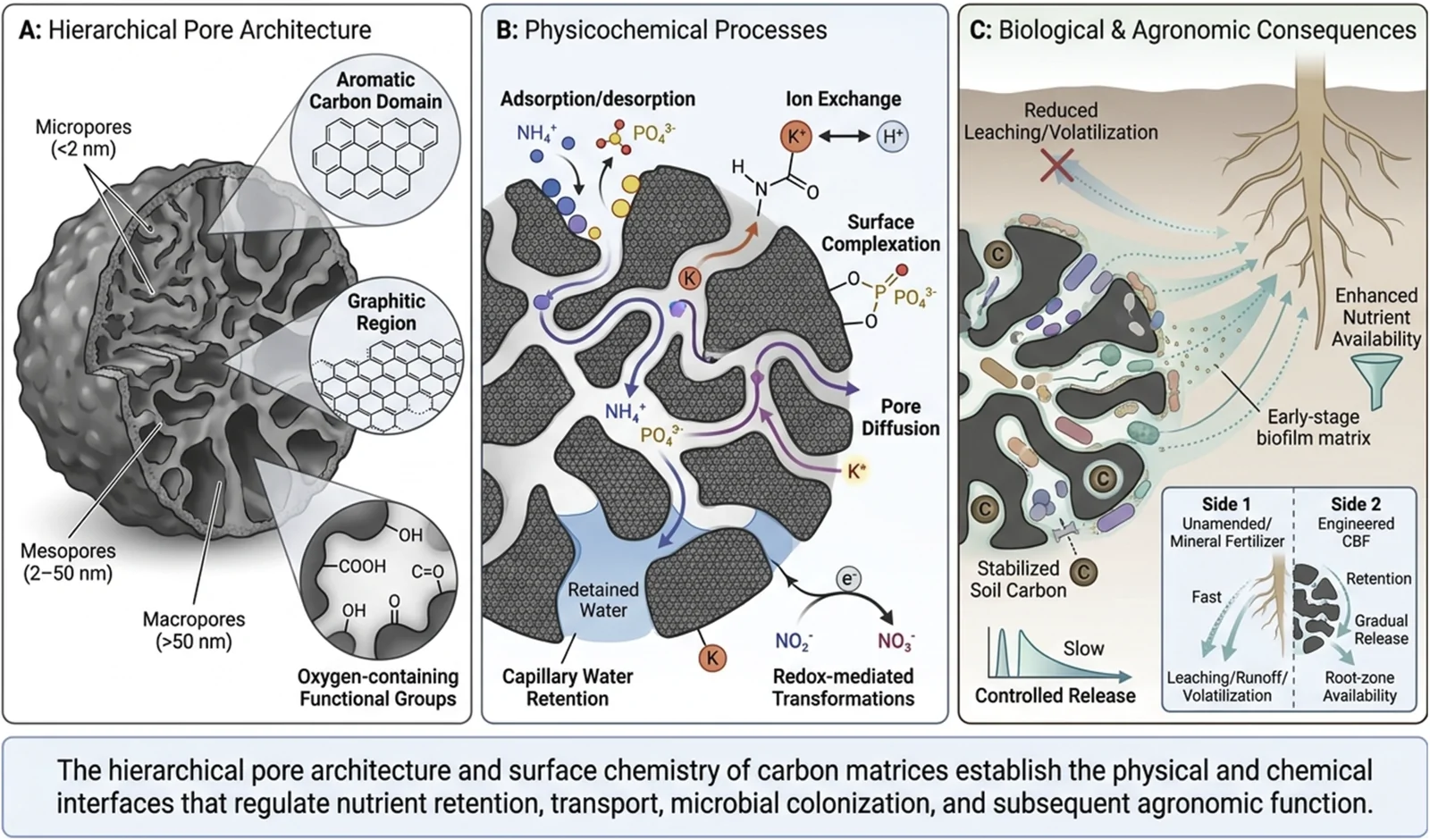
Hierarchical carbon architecture governing nutrient retention, transport, and soil–microbial function. (A) Hierarchical pore architecture of the carbon matrix, showing micropores, mesopores, and macropores, together with aromatic carbon domains, graphitic regions, and oxygen-containing functional groups. (B) Physicochemical processes, including adsorption/desorption, ion exchange, surface complexation, pore diffusion, capillary water retention, and redox-mediated transformations. (C) Biological and agronomic consequences, including reduced leaching/volatilization, early-stage biofilm formation, stabilized soil carbon, controlled nutrient release, and enhanced nutrient availability. Together, micropores, mesopores, and macropores provide distinct physicochemical and biological microsites, while surface functional groups and charge characteristics regulate adsorption, ion exchange, complexation, water retention, and nutrient diffusion. These properties collectively influence microbial habitat formation, controlled nutrient delivery, nutrient-use efficiency, and soil carbon stabilization (Fig. 2 partly generated by Google Gemini and figure Labs).

### 4.2. Nutrient enrichment and functionalization strategies

Building on the structural characteristics of carbon matrices, nutrient enrichment and surface functionalization provide the primary means of converting carbon materials into engineered fertilizer platforms. Nutrient impregnation enables macro- and micronutrients to be physically or chemically loaded onto carbon matrices, while co-pyrolysis can incorporate nutrients during carbon formation and promote their retention within the resulting matrix.^74^ Mineral coatings or surface precipitation with nutrient-bearing phases, such as calcium phosphates and magnesium compounds, can further regulate nutrient dissolution and release. Chemical functionalization, including oxidation, activation, and surface-group modification, can alter reactive sites and surface charge, thereby improve nutrient binding and enabling more controlled nutrient release.^75^ In parallel, microbial immobilization introduces beneficial microorganisms, such as plant-growth-promoting rhizobacteria and phosphate-solubilizing bacteria, into the carbon matrix, using its porous structure as a protective carrier and habitat. These strategies can be applied individually or in combination to develop multifunctional CBFs with improved nutrient retention, controlled delivery, and microbial support.^76^ Importantly, the engineering strategy determines the functional performance of the final CBF, linking carbon-matrix properties with nutrient-use efficiency and rhizosphere functionality.^77^Fig. 3 outlines the physical, chemical, and biological processing strategies used to convert raw carbon feedstocks into multifunctional rhizosphere delivery platforms. Unmodified biochar or hydrochar matrices initially lack targeted release kinetics and optimized surface charges, necessitating specific functionalization pathways to tune their performance. The framework categorizes five distinct engineering strategies, explicitly distinguishing between non-biological matrix alterations and microbial inoculation methods. Abiotic modification strategies include nutrient impregnation *via* pressure-assisted pore loading, co-pyrolysis of biomass with mineral precursors to embed nutrients within the aromatic carbon framework, mineral shell encapsulation to regulate dissolution rates, and chemical surface functionalization to introduce oxygenated groups (–OH, –COOH, >C=O) that alter surface charge density. In contrast, biological functionalization relies on microbial immobilization, loading protective macropores with plant growth-promoting rhizobacteria (PGPR) and phosphate-solubilizing bacteria (PSB). When introduced into the soil environment, these engineered CBFs deliver targeted nutrients directly to root surfaces, enhance localized ion retention, foster rhizospheric microbial activity, and prevent environmental leaching losses.

**Fig. 3:**
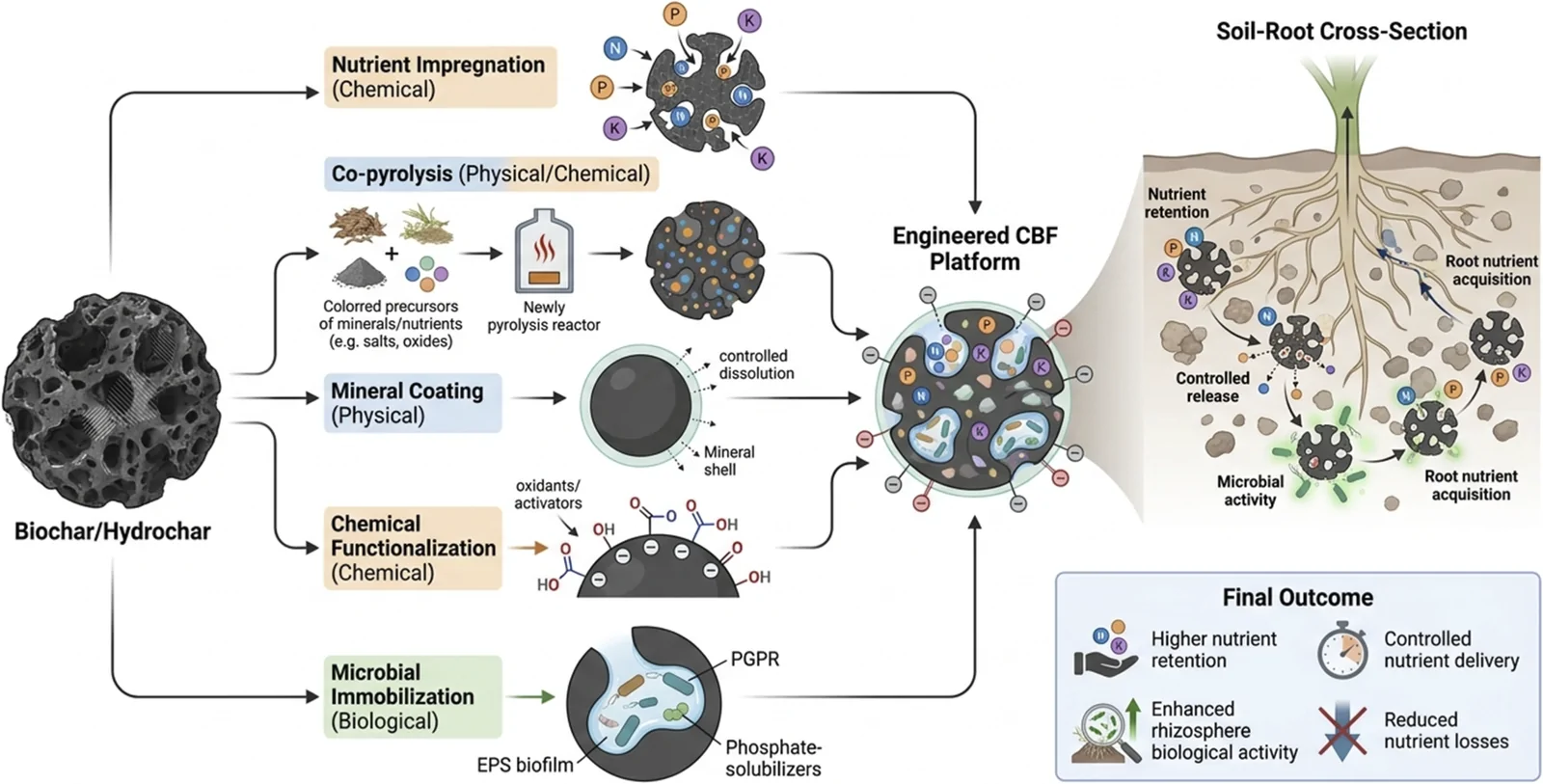
Synthesis pathways and functionalization strategies for engineering carbon-based fertilizers (CBFs) to enhance rhizosphere performance. Five primary modification strategies, nutrient impregnation, co-pyrolysis, mineral coatings, chemical functionalization (–OH, –COOH, >C=O), and microbial immobilization (PGPR, PSB), transform raw carbon matrices into multifunctional delivery systems. Black horizontal arrows trace raw material processing to final field performance; colored output arrows denote nutrient retention (cyan), controlled delivery (yellow), microbial proliferation (green), and suppressed leaching (red/crossed drop icon). Abbreviations: CBF, carbon-based fertilizer; PGPR, plant growth-promoting rhizobacteria; PSB, phosphate-solubilizing bacteria; N/P/K, nitrogen/phosphorus/potassium (Fig. 3 partly generated by Google Gemini and figure Labs).

### 4.3. Soil–plant–microbe functions of engineered carbon matrices

The functionalized carbon matrix acts as an interface linking nutrient delivery with soil biological processes and plant nutrient acquisition. Nutrient-loaded carbon matrices can regulate the retention, transformation, and gradual release of nutrients, thereby improving their availability in the rhizosphere while reducing losses through leaching and volatilization.^74^ At the same time, carbon surfaces and pore networks can provide microsites that support the persistence and activity of beneficial microorganisms.^78^ Deliberate immobilization of beneficial microorganisms within engineered carbon matrices significantly enhances microbial survival and persistence in adverse soil environments.^79^ These structured platforms enable the precise regulation and effective utilization of beneficial inoculants to foster synergistic plant–microbe interactions.^80^ Consequently, these microbially mediated transformations, particularly biological nitrogen cycling and phosphorus solubilization, drive targeted nutrient mobilization and optimize root acquisition efficiency.^81,82^

The resulting changes in the rhizosphere can influence root development and plant physiological performance. Improved nutrient availability and microbial activity may promote root elongation, lateral branching, and root-hair development, thereby increasing the effective root surface area for nutrient acquisition.^83^ Microbial–carbon–plant interactions can additionally influence phytohormonal signaling and plant metabolic processes, including antioxidant activity and stress-responsive pathways.^84^ Consequently, engineered carbon matrices can function beyond passive nutrient reservoirs by coordinating nutrient availability, microbial activity, and plant responses within the rhizosphere. This integrated soil–plant–microbe interface provides the mechanistic basis for enhanced nutrient-use efficiency and improved crop performance under variable environmental conditions.^85^

### 4.4. Carbon-matrix performance under environmental stress

The functional benefits of engineered carbon matrices extend beyond nutrient delivery to improving soil and plant performance under environmental stress. By modifying water retention, nutrient availability, and rhizosphere biological activity, CBFs can help buffer the effects of drought, salinity, and temperature fluctuations.^86^ Under water limitation, porous carbon matrices can improve soil moisture retention and maintain more favorable conditions for root water uptake, while sustained nutrient availability supports plant growth during periods of restricted resource supply.^87^ Under saline conditions, carbon-mediated changes in ion retention and soil chemical buffering may reduce the availability and uptake of potentially toxic ions while improving the retention of essential nutrients. Carbon-associated microbial habitats can further support beneficial microorganisms that facilitate nutrient cycling and alleviate stress.^88^ Collectively, these interactions can contribute to improved root development, nutrient acquisition, and physiological stability under abiotic stress. However, the magnitude and direction of these effects depend strongly on carbon feedstock, production conditions, application rate, soil properties, climate, and crop genotype.^12^ Therefore, environmental-stress performance should be evaluated as a context-dependent outcome of the integrated carbon–soil–microbe–plant system rather than as an intrinsic property of CBFs.^89^

### 4.5. Mechanisms governing carbon-based fertilizer function

Nutrient retention and release from carbon-based fertilizers are governed by several complementary but mechanistically distinct processes that depend on the structural and surface characteristics of the carbon matrix.^10^ Adsorption–desorption primarily involves reversible interactions between nutrient species and reactive surface sites, with surface area, pore accessibility, aromatic domains, and oxygen-containing functional groups influencing binding strength and reversibility.^90^ Ion exchange is governed by the density and nature of charged surface sites and the cation- or anion-exchange capacity of the carbon matrix, thereby regulating the reversible retention of ionic nutrients.^91^ Pore diffusion governs nutrient transport through the internal pore network and is influenced by pore-size distribution, connectivity, tortuosity, and water accessibility. In contrast, redox reactions involve electron-transfer processes associated with redox-active surface moieties and can alter the chemical speciation, mobility, and bioavailability of certain nutrients.^92^ Microbial nutrient transformation represents a biologically mediated pathway in which microorganisms associated with carbon surfaces and pores mineralize, solubilize, immobilize, or transform nutrients, thereby modifying their availability to plants.^93^ Thus, carbon architecture establishes the physical and chemical environment within which these processes occur: surface chemistry regulates adsorption and redox reactivity, surface charge controls ion exchange, pore structure governs diffusion, and microbial habitat supports biologically mediated nutrient transformation. The relative contribution of each mechanism is further influenced by soil pH, moisture, ionic strength, nutrient concentration, and microbial activity, collectively determining the overall nutrient-release behavior and agronomic performance of CBFs.^94^

Microbial mediated nutrient transformations are another very important mechanism. The porous carbon matrices respectively provide a habitat for PGPR, phosphate-solubilizing bacteria and other beneficial soil microbes. These microorganisms contribute to nitrogen fixation, phosphorus solubilization and decomposition of organic matter and thus release nutrients in a regulated way that is under biological control.^95^

Environmental and stimuli-responsive mechanisms allow CBFs to release nutrients in an adaptive way in response to soil moisture, pH and temperature.^96^ Increased soil moisture accelerates diffusion; acidic or alkaline soils promote ion desorption, which in turn depends on pH. Periodic temperature changes throughout the season regulate microbial activity and nutrient mineralization.^97^ These environmentally responsive characteristics make carbon-based fertilizers “smart” nutrient systems, which can balance nutrient release with plant demand and changing soil conditions, thereby creating nutrients with high nutrient use efficiency or low environmental impacts.^98^

Collectively, these mechanisms operate in an interconnected manner, with carbon-matrix architecture controlling nutrient accessibility and transport, surface charge governing ion exchange, and microbial activity contributing to biologically mediated nutrient transformation. Their relative importance depends on carbon feedstock, processing conditions, surface chemistry, soil physicochemical properties, and environmental conditions. Thus, the agronomic performance of CBFs arises from the interaction of physical retention, chemical exchange, transport, and biological transformation processes rather than from any single mechanism.^99^

Fig. 4 presents the carbon-based fertilizer as an active interface between the soil environment, rhizosphere microorganisms, and plant roots. The physicochemical side of the framework illustrates how adsorption, ion exchange, pore diffusion, surface interactions, redox processes, and condition-dependent desorption regulate nutrient retention and movement within the carbon matrix. In parallel, the biological component depicts PGPR- and PSB-associated processes, including microbial colonization, nutrient transformation, and root-associated signaling. The lower component connects these processes with environmental stress buffering, particularly through water retention under drought conditions and immobilization or attenuation of potentially toxic ions under salinity stress. Together, the Fig. 4 emphasizes that carbon-based fertilizer performance emerges from the interaction between material properties and biological processes rather than from nutrient retention alone.

**Fig. 4:**
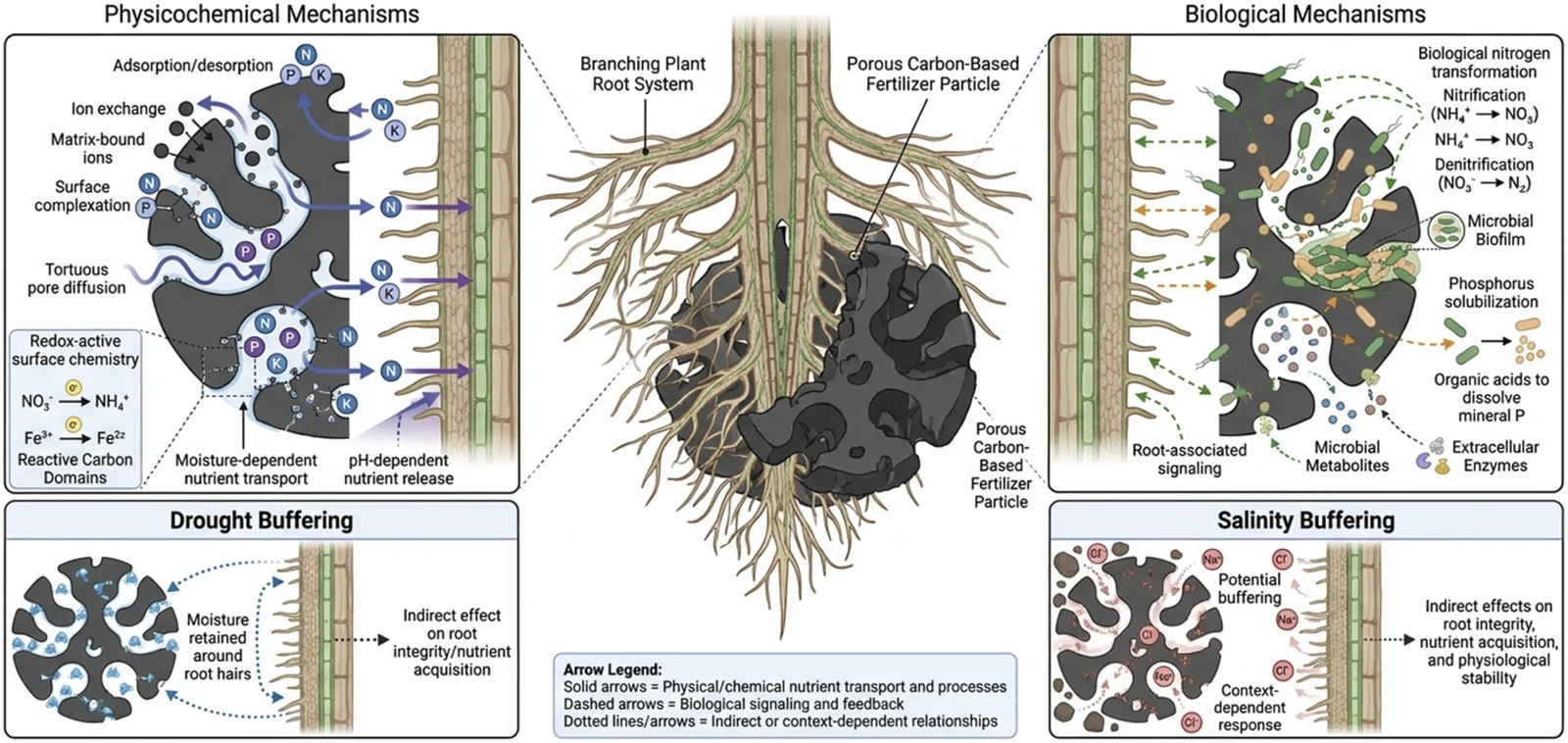
Integrated soil–plant–microbe interface governing physicochemical nutrient dynamics and biological stress buffering. Carbon matrices regulate nutrient adsorption, ion exchange, pore diffusion, redox reactions, and condition-dependent release while providing microsites for beneficial microorganisms. Microbial nutrient transformations and root-associated signaling contribute to nutrient acquisition and plant responses under environmental stress (Fig. 4 partly generated by Google Gemini and figure Labs).

### 4.6. Molecular surface chemistry and redox mechanisms

Beyond the general mechanisms of adsorption, ion exchange, diffusion, and microbial transformation, the chemical behavior of carbon-based fertilizers is strongly influenced by molecular interactions at the carbon–solution interface. Aromatic domains can participate in π–π electron donor–acceptor interactions with compatible aromatic or conjugated species, whereas oxygen-containing functional groups, including carboxyl, hydroxyl, phenolic, and carbonyl groups, provide sites for hydrogen bonding, electrostatic interactions, and Lewis acid–base interactions.^100,101^ Nutrient ions can also interact with carbon surfaces through outer-sphere or inner-sphere complexation. Outer-sphere complexes retain the hydration shell of the adsorbed ion and are predominantly stabilized by electrostatic interactions, whereas inner-sphere complexes involve more direct coordination or ligand exchange with surface functional groups or mineral-associated sites. The relative contribution of these interactions depends on surface functional-group density, carbon oxidation state, mineral composition, nutrient speciation, pH, and ionic strength.^101^

The degree of carbonization and graphitization further influences the electronic properties and redox behavior of carbon matrices.^102^ Condensed aromatic and graphitic domains can facilitate electron transport, while redox-active functional groups, including quinone/hydroquinone- and phenolic-type moieties, can participate in reversible electron-transfer reactions. Consequently, some carbon matrices may function as electron donors, acceptors, or electron shuttles in soil environments.^103^ These properties can influence redox-sensitive nutrient transformations, including nitrogen turnover and the dissolution or precipitation of Fe- and Mn-associated nutrient phases. However, electron-shuttling capacity should not be considered an intrinsic property of all biochars or carbon-based fertilizers; it depends on feedstock, thermal processing, degree of graphitization, surface functionalization, mineral constituents, and environmental redox conditions. The resulting effects on nutrient cycling are therefore material- and soil-specific.^104^

These molecular interactions provide a mechanistic link between carbon-matrix properties and nutrient availability in the rhizosphere. Strong inner-sphere coordination may promote nutrient retention, whereas weaker outer-sphere interactions can facilitate reversible exchange and release. Similarly, redox-active carbon domains may alter nutrient speciation or the reactivity of associated mineral phases, thereby modifying nutrient mobility and bioavailability.^105^ The final outcome depends on the balance between surface binding, desorption, electron transfer, microbial transformation, and plant nutrient uptake. Accordingly, molecular surface chemistry should be considered an underlying determinant of CBF performance rather than an independent mechanism operating separately from physical and biological processes.^106^

## 5. Carbon-based fertilizer as rhizosphere engineering system

CBFs have recently been recognized not only as a carrier of nutrients, but also as rhizosphere engineering systems able to restructure the biological, chemical, and physical processes at the root–soil interface.^107^ The rhizosphere is a highly dynamic microenvironment where plant roots, soil microbes, and organic and mineral components of the soil interact to regulate nutrient availability and plant physiological responses.^108^ CBFs affect the microbial colonization, nutrient cycling pathways and plant metabolic signaling networks. Consequently, carbon-based fertilizers are an emerging strategy for precision rhizosphere management of hitherto poorly modulated plant regulators, with the goal of deliberately designing soil carbon structure to optimize microbial activity, nutrient dynamics, and plant resilience under environmental stress.^109^

### 5.1. Carbon–root–microbiome interactions

A major mechanism through which CBFs can influence plant productivity is the regulation of the soil–plant–microbe interface through both direct physicochemical effects of the carbon matrix and microbially mediated processes. Carbon matrices derived from biochar or hydrochar possess porous architectures and diverse surface functional groups that can modify soil pH, moisture availability, nutrient retention, and microsite conditions, thereby directly influencing the rhizosphere environment.^110^ These direct effects should be distinguished from responses that arise through changes in microbial abundance, activity, and community composition. The porous structure can additionally provide protected microsites that reduce microbial exposure to environmental stresses such as desiccation and fluctuations in nutrient availability, thereby supporting microbial persistence in the rhizosphere.^111^

When beneficial microorganisms are intentionally incorporated, carbon matrices can also function as microbial carriers rather than merely passive soil amendments. Their interconnected pores and reactive surfaces can facilitate microbial attachment, retention, and protection, potentially improving the persistence and establishment of plant PGPR and other beneficial microorganisms. These microorganisms may include nitrogen-fixing bacteria, phosphate-solubilizing microorganisms, and siderophore-producing strains that contribute to plant nutrition through nitrogen fixation, nutrient solubilization, phytohormone production, and pathogen suppression.^112^

Thus, the carrier function represents a microbially mediated pathway that depends on the compatibility between the carbon matrix's properties and the physiological requirements of the introduced microorganisms. The carbon matrix can further support microbial consortia and biofilm formation, facilitating interactions among microorganisms and potentially enhancing metabolic cooperation and nutrient transformation.^113^ Biofilms consist of microbial communities embedded within extracellular polymeric substances attached to carbon surfaces, where microorganisms can exchange metabolites and coordinate functional activities through cell-to-cell signaling.^114^ Accordingly, carbon materials may serve as physical scaffolds that facilitate microbial organization and persistence, whereas subsequent nutrient mineralization, solubilization, and transformation are primarily microbially mediated processes. These processes can ultimately enhance nutrient availability and plant nutrient acquisition.^115^ Beyond their carrier function, CBFs can indirectly modify rhizosphere microbial communities by altering soil physicochemical conditions, including pH, redox potential, moisture retention, and organic carbon availability.^19^ Such environmental shifts may favor functionally important microbial groups, including *Actinobacteria*, *Proteobacteria*, and *Firmicutes*, which participate in nutrient cycling and plant–microbe interactions. Therefore, the effects of CBFs on rhizosphere microbiomes arise from a combination of direct modification of the soil habitat and subsequent microbial responses, rather than from a single biochar-mediated mechanism. Collectively, these interactions can strengthen nutrient cycling, plant–microbe associations, and rhizosphere functioning.^116^

### 5.2. Regulation of nutrient cycling processes

CBFs affect biogeochemical nutrient cycling in the rhizosphere through both direct physicochemical effects of the carbon matrix and microbially mediated nutrient transformations. In nitrogen cycling, biochar can directly retain ammonium and nitrate through adsorption and ion exchange, thereby reducing their leaching and volatilization losses.^117^ In contrast, microorganisms regulate nitrogen transformations through ammonification, nitrification, denitrification, and, where applicable, biological nitrogen fixation, while the porous carbon matrix can provide a favorable habitat for these functional microbial groups.^118^ Recent research has shown that biochar-amended soils can improve nitrogen retention and nitrogen-use efficiency through the complementary effects of physicochemical nutrient stabilization and microbial regulation of nitrogen transformations.^119^

Phosphorus cycling similarly involves distinct but complementary contributions from biochar and microorganisms. Phosphorus in soil is often immobilized through precipitation or strong association with calcium, iron, and aluminum compounds, thereby limiting its availability to plants. Biochar can directly modify P availability by altering soil pH and interacting with phosphate-retaining mineral phases, whereas phosphate-solubilizing microorganisms actively mobilize poorly available P through organic acids, phosphatases, and chelating compounds.^120^ Thus, the direct physicochemical effects of biochar and the enzymatic and metabolic activities of microorganisms should be considered separate processes that can operate synergistically in CBF-amended soils.

A major emerging concept is carbon–nutrient coupling, referring to the interconnected cycling of carbon, nitrogen, and phosphorus in the rhizosphere.^121^ Carbon-based fertilizers introduce relatively persistent carbon structures that can modify microbial habitat and provide carbon substrates or electron-transfer interfaces, depending on carbon quality and soil conditions. This carbon input can stimulate microbial activity and influence nutrient turnover through mineralization and enzymatic processes.^122^ Simultaneously, the sorption capacity of carbon matrices can retain nutrients within the root zone, creating a complementary relationship in which biochar improves nutrient retention while microorganisms mediate nutrient transformation and mobilization. This integrated carbon–microbe–nutrient interaction can contribute to enhanced soil fertility and longer-term nutrient sustainability.^123^

### 5.3. Root architecture and plant metabolic responses

In addition to affecting microbial and nutrient dynamics, CBFs have direct impacts on plant root development and determination of metabolic regulation. Carbon-enriched soils often favor improved root growth in terms of root length, lateral branching and root hair density. These changes in root architecture increase the amount of root surface area for uptake of nutrients and increase plant access to the soil resources.^124^ One important mechanism behind reliance by which the brains may influence their functioning are hormonal signaling pathways. Interactions between matrices that contain carbon, microbial communities, and plant roots may be stimulated in the production of phytohormones such as auxins, cytokinins, and gibberellins.^125^

Many PGR associated with carbon materials produce indole-3 – acetic acid (IAA), which enhances root elongation and branching. In addition, the amendments based on carbon can modulate the ethylene and abscisic acid signaling pathways and, as such, play a role in determining the plant response to environmental stress.^126^ CBFs also increase enzyme activation and regulate metabolism in plants. Increased activity of enzymes, *e.g.*, nitrate reductase, phosphatases, and antioxidant enzymes, has been observed in plants grown in carbon-amended soil. These responses by enzymes allow for greater assimilation efficiency of nutrients, and boost plant defense mechanisms against oxidative stress.^127^

Moreover, carbon-based fertilizers can induce the accumulation in plants of protective metabolites, such as phenolics, flavonoids and osmolytes which help the plants to deal with drought, salinity and heavy metal stress.^128^ Another more recently recognized mechanism is through activation of stress – signaling pathways. Carbon materials may affect gene expression of plants involved in stress tolerance such as reactive – oxygen – species detoxification genes, osmotic adjustment, and biosynthesis of secondary metabolites.^129^ Through these mechanisms, CBFs are not only able to improve nutrient uptake, but also to increase the physiological relic of the plant in adverse environmental conditions.

### 5.4. Engineered rhizosphere carbon interface

The combinations of microbial, chemical and plant physiological processes lead to the development of the concept of the engineered rhizosphere carbon interface, where carbon-based fertilizers are multifunctional platforms that control soil, microbes and plants interaction.^109^ In this framework, carbon matrices are intentionally fabricated with certain physicochemical characteristics for transcription of a given rhizosphere performance (*i.e.*, pore organization, surface functionality, nutrient loading, *etc.*) to maximize rhizosphere performance.^107^

Functional groups on the carbon surfaces help the uptake of nutrients and their controlled release, and through microbial biofilm, nutrients are converted to plant-available forms. This coordination system is more effective at nutrient use efficiency, reduces environmental losses and is capable of sustainable soil fertility.^130^ Recent developments in materials science and soil microbiology have paved the way for new opportunities to design next generation smart carbon fertilizer and combine nanotechnology, microbial inoculants and nutrient engineering. Such systems can be designed to respond to the statuses of soil, plant development, and environmental stresses so that they enable precision management of the rhizosphere.^131^Fig. 5 presents a comprehensive tri-zonal conceptual model that integrates the physical, chemical, and biological mechanisms CBFs within the rhizosphere engineering framework. The central anchor illustrates a porous biochar particle tightly intertwined with lateral roots and dense root hairs, serving as the spatial foundation for both abiotic chemical reactions and biotic colonization.

**Fig. 5:**
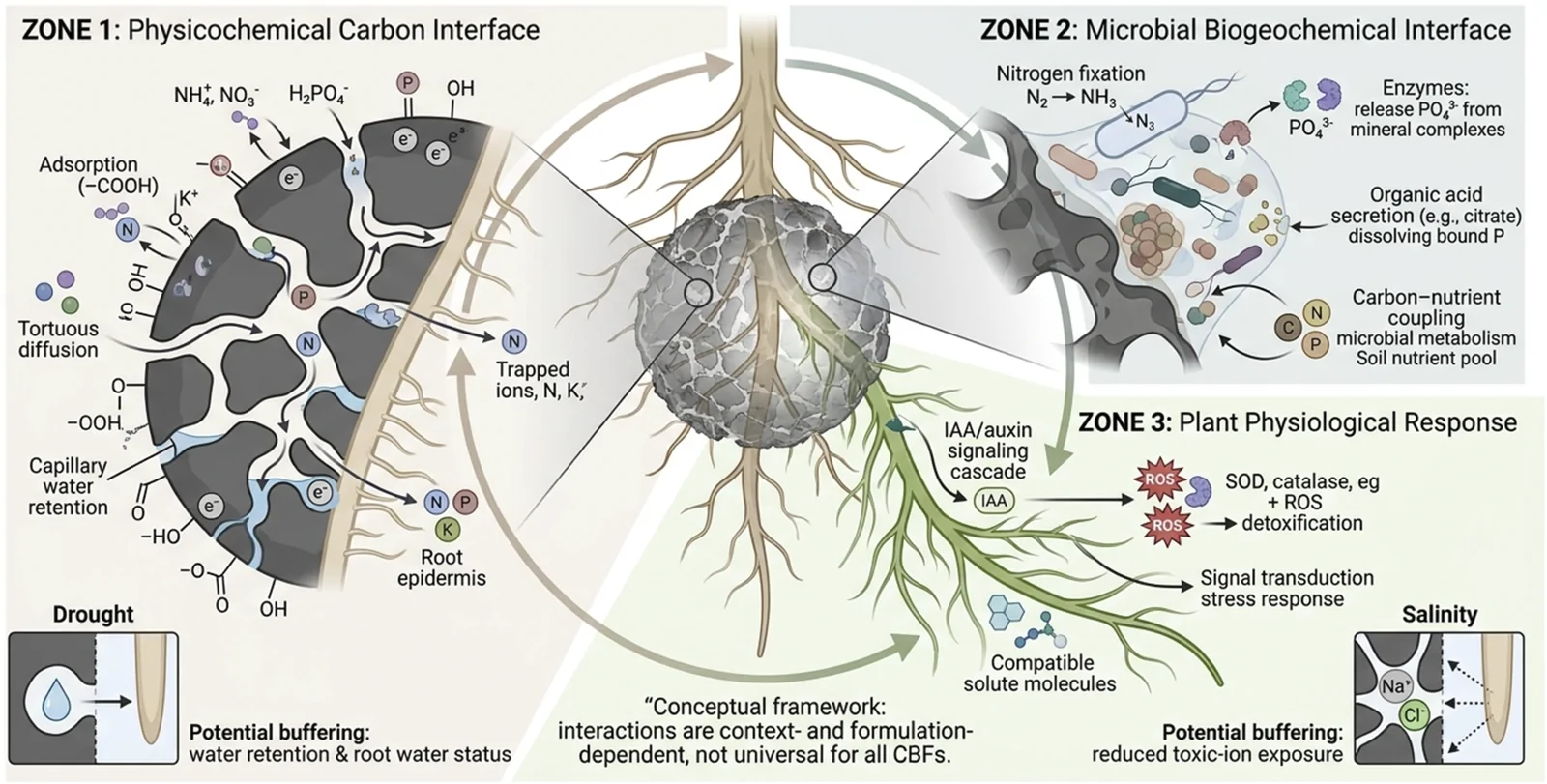
Tri-zonal rhizosphere engineering model of carbon-based fertilizers. The conceptual framework integrates three interconnected zones: (1) physicochemical processes at the carbon–soil interface, including nutrient retention, ion exchange, diffusion, and redox interactions; (2) microbial processes within protected carbon microsites, including nitrogen transformation and phosphorus solubilization; and (3) plant responses involving root architectural modification, hormonal signaling, antioxidant activity, and stress-related physiological adjustment (Fig. 5 partly generated by Google Gemini and figure Labs).

Zone 1 delineates the non-biological physicochemical matrix dynamics that occur directly on the carbon carrier surface and within its internal structure. Surface functionalization with carboxyl (–COOH) and hydroxyl (–OH) groups establishes reversible ion exchange sites that bind essential nutrient species, including ammonium (NH_4_^+^), nitrate (NO_3_^−^), and phosphate (PO_4_^3−^), thereby preventing passive leaching losses. Concurrently, tortuous internal pore channels control capillary moisture retention and passive fluid diffusion, while surface electron-transfer moieties (e^−^) provide localized pH and redox buffering to stabilize the surrounding chemical microenvironment.

Zone 2 isolates the microbially mediated biogeochemical cycling occurring within protected matrix microsites. Internal macropores act as protective sanctuaries for plant growth-promoting rhizobacteria (PGPR) and phosphate-solubilizing bacteria (PSB), facilitating their encapsulation within extracellular polymeric substance (EPS) biofilms that shield them from environmental desiccation. These sheltered microbial communities actively drive biological nitrogen fixation, organic acid secretion, and enzymatic phosphorus solubilization. This establishes a tight carbon–nutrient coupling loop, wherein biochar carbon substrates fuel continuous microbial turnover and sustained soil biological fertility.

Zone 3 illustrates the downstream physiological, morphological, and stress-buffering outcomes expressed by the host plant. Compared to standard development in unamended soils, CBF addition induces significant root morphological remodeling, characterized by accelerated elongation, increased lateral branching, and higher root hair density that dramatically expands uptake surface area. Chemically, signal transduction pathways up-regulate auxin (IAA) production, boost antioxidant enzyme activation for reactive oxygen species (ROS) scavenging, and stimulate protective osmolyte accumulation (flavonoids and phenolics). The embedded stress resilience panel highlights how these combined mechanisms mitigate abiotic stressors: during drought, capillary water retention in carbon pores maintains root turgor, whereas under saline conditions, the matrix physically immobilizes toxic sodium (Na^+^) and chloride (Cl^−^) ions away from sensitive root tissues.

## 6. Nano-enabled smart carbon fertilizers

In this review, “smart” carbon-based fertilizers refer specifically to engineered systems in which material properties or functional components enable controlled or stimulus-responsive regulation of nutrient availability; the term is not used to imply autonomous sensing, decision-making, or universally validated field performance. Recent advances in nanotechnology and materials engineering have accelerated the research and development of nano-enabled carbon fertilizers, which represent a new generation of stimuli-responsive nutrient delivery systems targeting an increase in nutrient use efficiency and control of by rhizosphere related processes and decrease the environmental losses.^132^ Conventional carbon-based fertilizers *e.g.* biochar, compost *etc.* play the main role in the soil conditioner and nutrient reservoir. Nevertheless, nanoscale carbon materials present distinctive physicochemical properties, including increased surface reactivity, tunable functional groups, and size-dependent surface and electronic characteristics, which may enable more precise regulation of nutrient dynamics and plant–soil interactions.^11^ However, these properties should not be interpreted as direct evidence of specific agricultural mechanisms; their functional significance depends on the material characteristics and experimental system. Nano-enabled carbon fertilizers have therefore started to be considered multifunctional platforms in which nutrient delivery, surface-mediated interactions, and potentially redox-associated processes can be integrated. These systems represent an emerging approach to sustainable agriculture in which nanostructured carbon materials are designed to balance nutrient availability with physiological demand of plants and reduce the carbon footprint of fertility practices.^133^

### 6.1. Nano-biochar and carbon nanomaterials

Among the best middles in this regard is the emergence of nano – biochar, which is created by mechanical milling, chemical activation, or new thermal processing training of traditional biochar materials. Compared to bulk grade biochar, nano-amino biochar has a significantly higher surface area, smaller particle size and higher surface reactivity properties. These attributes can enhance nutrient adsorption and increase opportunities for interactions with soil microorganisms and root-associated environments, although the magnitude of these effects depends on particle characteristics and soil conditions.^134^ Nano-biochar particles may access smaller soil pore domains and establish close associations with root-associated environments, potentially facilitating nutrient interactions in the rhizosphere. Such interactions should not, however, be interpreted as evidence of direct penetration into plant cells unless supported by appropriate experimental evidence. In addition to nano bioactive (nano – biochar), a wide variety of engineered nanomaterials of carbon are being explored for agricultural applications.^135^

Carbon dots (CDs), a class of nanoscale carbon particles typically for sizes less than 10 nm, are of interest due to their photoluminescent properties and potential biointegration. These materials have the advantage of acting as carriers for micronutrients or bioactive molecules, and at the same time may influence other physiological processes of the plant such as photosynthesis and antioxidant metabolism. Carbon dots have been associated with changes in nutrient transport and plant growth under stress conditions, although the underlying mechanisms remain dependent on material properties and plant systems.^136^

Another important category of materials includes those obtained from graphene, *i.e.* graphene oxide (GO) and reduced graphene oxide (rGO). Compared to conventional materials, these are materials with exceptional surface area, electrical conductivity and mechanical stability. Within the soil systems, graphene-based materials can be used to adsorb nutrient ions, interact with microbial cells and control redox reactions responsible for nutrient transformations.^137^ Functionalized nano-biochar composites drive carbon nanomaterials towards further development in the agricultural sector. By incorporating metal oxides, clay minerals or polymer coatings with nano-biochar, researchers have created nano-biochar systems that are able to deliver macronutrients and micronutrients in a controlled manner.^135^ These engineered nanomaterials with carbon components provide multifunctional capabilities such as nutrient adsorption capability, microbial support capability, pollutants immobilization capability, and enhanced soil structural stability.^32^

### 6.2. Smart nutrient delivery systems

Nano-enabled carbon fertilizers are gradually developed into controlled nutrient delivery system, which can release nutrients in response of environmental stimuli or plant physiological signals.^138^ Stimuli-responsive release can arise from chemically defined changes in the carrier matrix, including protonation–deprotonation of ionizable functional groups, pH-dependent changes in surface charge, swelling of responsive polymer coatings, or cleavage of labile chemical bonds.^139^ For example, carboxyl-containing surfaces can undergo protonation or deprotonation with changes in soil pH, altering electrostatic interactions with nutrient ions, whereas amino-containing polymers can change their charge state and swelling behavior under different pH conditions.^140^ Similarly, enzyme-responsive systems can incorporate biodegradable or enzyme-cleavable linkages whose hydrolysis increases matrix permeability and promotes nutrient release. Thus, the stimulus does not simply “trigger” nutrient release conceptually; it produces a defined chemical or structural change in the carrier that modifies nutrient binding, diffusion, or matrix degradation. The magnitude and direction of these responses depend on the composition of the carbon matrix, functionalization strategy, nutrient chemistry, and soil environment.^141^ Importantly, these mechanisms should be distinguished from autonomous or adaptive nutrient delivery: a system should be considered “smart” only when a defined environmental or biological stimulus produces a reproducible and experimentally demonstrated change in nutrient-release behavior.

Moisture- and temperature-responsive release can additionally regulate nutrient transport through changes in water accessibility, polymer swelling, diffusion coefficients, and microbial activity. Increased soil moisture can enhance water penetration into porous carbon matrices and accelerate nutrient diffusion through interconnected pores, whereas drying can restrict diffusion and reduce nutrient mobility.^142^ Temperature can similarly alter molecular diffusion and the activity of microorganisms and extracellular enzymes involved in nutrient transformation. These responses are therefore primarily physical or biological rather than evidence of an autonomous molecular “smart gate,” and their contribution should be interpreted according to the specific material and soil conditions.^143^ Such environmental responsiveness can nevertheless contribute to temporal regulation of nutrient availability under fluctuating rhizosphere conditions.^144^ Accordingly, moisture- and temperature-dependent responses should be interpreted as condition-dependent changes in nutrient transport or transformation rather than as evidence of autonomous sensing or decision-making by the fertilizer system.

Another strategy is targeted nutrient transport, in which engineered carbon materials are designed to improve nutrient retention, mobility, and delivery within the rhizosphere. Their small particle size, high surface reactivity, and functionalized interfaces can facilitate interactions with root exudates, soil minerals, and microbial metabolites, potentially modifying nutrient mobility and availability near plant roots.^145^ However, association of carbon nanomaterials with root surfaces should not be interpreted as direct cellular uptake or intracellular nutrient delivery unless demonstrated by appropriate localization, imaging, or tracer studies. Accordingly, current evidence more strongly supports the role of these materials in regulating nutrient availability and transport within the soil–root interface than a generalized mechanism of direct intracellular delivery. Such approaches may contribute to condition-responsive nutrient management by regulating nutrient retention, release, and availability near the root zone.^146^ Conventional fertilizers often have low nutrient utilization efficiency, resulting in substantial losses through leaching, volatilization, and runoff. Nano-enabled carbon fertilizers may address some of these limitations through controlled nutrient release, improved soil retention, and enhanced nutrient availability; however, their agronomic advantages should be distinguished from laboratory-scale proof-of-concept results and require validation under heterogeneous field conditions.^147^

A clear distinction is therefore required between established CBF mechanisms and emerging delivery concepts. Adsorption–desorption, ion exchange, pore-mediated diffusion, surface interactions, and microbial nutrient transformation are established processes, although their magnitude varies with material and soil conditions.^148^ In contrast, highly adaptive nutrient delivery, targeted intracellular transport, and autonomous stimulus integration remain emerging concepts requiring further experimental validation. The practical performance of nano-enabled CBFs may also differ between controlled laboratory systems and field environments because of variations in soil heterogeneity, moisture, temperature, microbial communities, material aging, and nutrient transport.^149^ Therefore, laboratory-scale improvements in nutrient retention or plant growth should not be considered equivalent to demonstrated field-scale agronomic efficacy. Long-term, multi-location field studies are required to establish the reliability, environmental fate, economic feasibility, and agronomic benefits of these systems.

Fig. 6 illustrates the integration of nano-enabled carbon platforms into the rhizosphere system, representing the next technological phase of carbon-based fertilizers. Surrounding green circular arrows trace bi-directional plant–carbon signaling and microbe-to-plant feedback loops, where plant growth-promoting rhizobacteria (PGPR) hosted within the matrix synthesize plant growth regulators (PGR) and phytohormones, specifically indole-3-acetic acid (IAA), to direct plant metabolic activity. The four surrounding subpanels delineate the specific abiotic nanoscale mechanisms and biotic outcomes of the platform. In the upper panels, non-biological matrix properties are highlighted: the top-left subpanel demonstrates tunable surface chemistry, where high-density functional moieties (–OH, –COOH) govern targeted ion exchange and complexation with nutrient species (NH_4_^+^, PO_4_^3−^) and multivalent cations (Ca^2+^, Al^3+^). The top-right subpanel highlights quantum effects, showing how embedded carbon quantum dots (CDs) act as electron shuttles for redox mediation, driving key valence transitions such as iron reduction (Fe^2+^ → Fe^3+^) to enhance nutrient bioavailability. The lower subpanels details biological housing and controlled delivery functions. The bottom-left panel highlights the nano-porous matrix architecture, which creates protected microhabitats that shield PGPR and phosphate-solubilizing bacteria (PSB) from environmental desiccation and biological predation. Concurrently, the bottom-right panel depicts a stimuli-responsive release mechanism (historically referred to as a “smart gate”), where external environmental triggers, such as localized soil moisture shifts and temperature variations, regulate the gate kinetics, releasing primary nutrients (N, P, K) on demand to match crop uptake requirements. Table 2 summarizes the molecular mechanisms, carbon features, and chemical processes governing nutrient retention and stimuli-responsive release in carbon-based fertilizers.

**Fig. 6:**
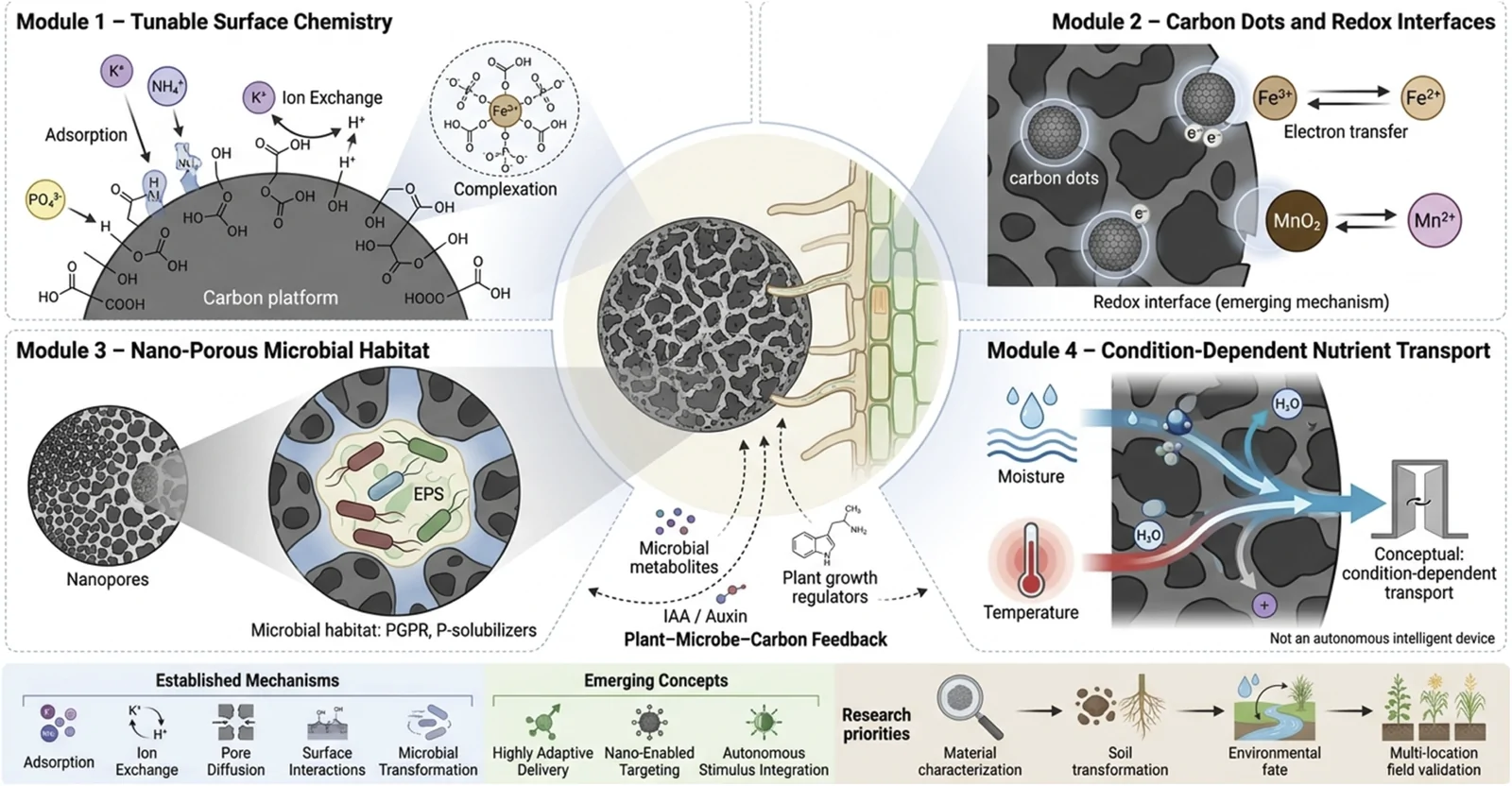
Nano-enabled stimuli-responsive carbon platforms governing rhizosphere signaling, electron transfer, and protective microhabitats. Four corner subpanels detail tunable surface chemistry (–OH, –COOH binding NH_4_^+^, PO_4_^3−^, Ca^2+^), quantum dot redox mediation (Fe^2+^/Fe^3+^ electron shuttling), nano-porous microbial shelters against desiccation, and stimuli-responsive gates triggered by moisture and temperature. Green circular arrows map bi-directional signaling pathways involving plant growth regulators (PGR) and auxin (IAA) surrounding the central root–carbon interface. Abbreviations: CD, carbon dots; IAA, indole-3-acetic acid; PGPR, plant growth-promoting rhizobacteria; PSB, phosphate-solubilizing bacteria; PGR, plant growth regulators (Fig. 6 partly generated by Google Gemini and figure Labs).

**Table 2:** Molecular mechanisms governing nutrient retention, transformation, and stimuli-responsive release in carbon-based fertilizers

Mechanism	Carbon/material feature	Chemical process	Nutrient consequence	References
π–π interaction	Aromatic/graphitic domains	Electron donor–acceptor interaction	Nutrient/organic molecule retention	150
Lewis acid–base interaction	O/N-containing functional groups	Donor–acceptor coordination	Enhanced surface binding	151
Outer-sphere complexation	Charged surface	Electrostatic association	Reversible nutrient retention	152
Ion exchange	Charged functional groups	Cation/anion exchange	Controlled nutrient release	153
Pore diffusion	Connected pore network	Concentration-gradient transport	Sustained nutrient delivery	154
pH response	–COOH/–OH/–NH_2_ groups	Protonation/deprotonation	pH-dependent nutrient release	139
Enzyme response	Enzyme-cleavable carrier/linker	Hydrolysis/biodegradation	Biologically triggered release	155
Redox response	Redox-sensitive bonds/groups	Reduction/oxidation	Stimulus-dependent release	156

### 6.3. Laboratory-to-field translation

Laboratory-to-field translation remains a critical challenge for carbon-based fertilizers, particularly for nano-enabled systems. Many reported improvements in nutrient retention, controlled release, microbial activity, and plant growth have been demonstrated under controlled laboratory, hydroponic, greenhouse, or pot conditions, where soil properties and environmental variables can be tightly regulated.^157^ In field environments, however, heterogeneous soil physicochemical properties, fluctuating moisture and temperature, complex microbial communities, root–soil interactions, material aging, aggregation, and nutrient transport can substantially modify carbon-matrix behavior.^70^ Consequently, performance observed under controlled conditions should not automatically be interpreted as equivalent to field-scale agronomic efficacy. Long-term, multi-season and multi-location field trials are therefore required to determine whether the proposed nutrient-retention and stimuli-responsive mechanisms remain effective under realistic agricultural conditions.^158^ Such studies should integrate nutrient-use efficiency, crop productivity, soil health, environmental fate and persistence, economic feasibility, and potential ecological risks to assess the practical applicability of nano-enabled CBFs.

## 7. Agro-environmental benefits of carbon-based fertilizers

CBFs are multifunctional soil amendments, and at the same time, improve soil fertility, increase crop productivity and minimize environmental impacts related to conventional fertilization. Their large surface area, porous structure and reactive functional groups promote the excellent adsorption of nutrients, increase soil aggregation and promote the development of microbial habitats. Consequently, CBFs are part of the solution to restoring soil health, enhancing nutrient use efficiency, carbon sequestration, nutrient losses and immobilizing pollutants, to support sustainable low carbon agricultural patterns.^159,160^

CBFs significantly boost soil health through a better physical structure, chemical buffering capacity and biological activity. Carbon matrices from biochar or compost build up soil aggregation, porosity and water infiltration and enhance cation-exchange capacity facilitating the ability of soils to hold essential nutrients, such as ammonium and potassium.^161^

The other major advantages are the enhancement of nutrient-use efficiency (NUE). Carbon matrices play a major role as a nutrient reservoir for the adsorption of ions like ammonium, nitrate and phosphate ions and their slow release through ion exchange processes and diffusion. This controlled release matches the nutrient availability to the plant demand, minimizes nutrient losses in fertilizer form and optimizes the nutrient uptake by the crop and also reduces the input requirements.^162^

CBFs are also important contributors to mitigation of climate change through carbon storage. Ultimately stable aromatic carbon structures in material things all the way for biochar stick for decades to centuries in soils which may thus augment soil organic carbon stocks while improving soil fertility and water retention. Moreover, they have a high sorption capacity which decreases nutrient losses into the soil and gaseous emissions such as nitrous oxide and ammonia which reduces environmental pollution.^163^

## 8. Carbon based fertilizers for climate-resilient agriculture

Climate change is increasing abiotic stresses in agricultural systems including drought, salinity, heat waves and erratic rainfall patterns which affect soil processes, plant physiology and crop productivity. Within such a context, CBFs are increasingly recognized as climatic adaptive soil amendments that can contribute to plant tolerance to the changing climatic conditions as well as to stabilization of soil ecosystems.^161^ Beyond the contribution to nutrients, CBFs alter the physical structure of soils, increase rhizosphere microbial activity, and affect the metabolic response of plants, which together increase resilience of the crop. Carbon-enriched soils have the potential to reduce environmental heterogeneity by enhancing soil moisture balance and nutrient cycling pathways along with microbial interaction and plant stress signaling pathways, making these kind of fertilizers promising tools for climate adaptive agricultural management.^164^

CBFs aid the mitigation of drought stress mostly through their porous carbon structure that stores water on the micro- and mesopores and slowly releases it during dry periods preserving the moisture availability in the rhizosphere. Improved soil moisture conditions increase deep root growth as well as root hydraulic conductivity, plants then manage to maintain water uptake under limited water supply. Additionally, carbon amended soils activate friendly microorganisms that synthesize types of osmoprotectants and phytohormones like abscisic acid and indole-3-acetic acid involved in the improvement of stomatal control, osmoregulation, and antioxidant capacities in plants.^165^

These fertilizers are also beneficial for adaptation to salinity and heat stress by better aggregation and higher cation exchange capacities and therefore reduce the impact of sodium toxicity, while increasing retention of essential nutrients. Functional groups of carbon surfaces are able to adsorb excess sodium ions for improved soil chemical balance and enhanced soil moisture conditions are helpful for buffering temperature changes around plant roots.^166^

Furthermore, CBFs enhance the water retention in soil as well as hydrological resilience through greater pore volume, better infiltration, and less surface runoff. Their interaction with organic matter in the soil stimulates the formation of stable aggregates which protect the soil carbon and maintain the soil moisture availability under varying climatic conditions.^167^ CBFs increase stress resistance through microorganisms *via* microbiome, like plant growth-promoting rhizobacteria, and mycorrhizal fungi as habitat providers. These microbes act on nutrient cycling, produce stress alleviating compounds and activate plant defense pathways allowing plants to withstand environmental stresses and maintain productivity under changing climatic conditions.^168^

## 9. Circular bioeconomy integration and low-carbon farming systems

CBFs play a key role in the circular bioeconomy, where the conversion of agricultural residues, organic wastes and biomass streams of industrial production into value added soil amendments takes place. This methodology encourages sustainable waste management, greater resource efficiency and reduced environmental footprint of agricultural production.^9^ By providing nutrients to soil in form of stable carbon-rich fertilizers, CBF technologies help close nutrient cycles and conflate soil fertility issues and low-carbon and climate-smart agricultural systems, both for environmentally sustainable purposes and for economic value creation.^169^

CBFs are generally produced through waste-to-fertilizer valorization routes that use biomass either as the raw material of choice from diverse origin such as crop residues, livestock manure, waste from the food processing (FA), and organic matter municipal waste.^170^ Through thermochemical and biological procedures-such as pyrolysis, composting, hydrothermal carbonization and anaerobic digestion-well as through end uses, people in the wallets are designed biochar, fertilizers from compost production, amendments applied to digestate or hybrid carbon-nutrient structures. This approach to recycling reduces the environmental standard burdens associated with disposing of biomass waste whilst also recovering nutrients that would otherwise be lost reducing the need to spend on non-renewable mineral fertilizers.^171^

A bonus about CBFs is their ability to lower the carbon footprint. Conventional fertilizer production, in particular nitrogen production by the Haber–Bosch process, is a source of intensive energy consumption and a significant source of greenhouse gas production.^172^ In contrast, CBFs use renewable feedstocks from biomass and have the ability to sequester stable carbon in soils, as well as to improve nutrient retention and reduce nitrous oxide emissions to the environment to support low emission agricultural systems.^173^

CBFs are also compatible with carbon market and climate-policy frameworks, because stable carbon -products, such as biochar, can be used to act as long -term carbon sinks. There may be financial incentives offered to farmers to integrate their application into such climate mitigation programs if they adopt practices using carbon-based fertilizers.^174^

At the farm level, there is a dependency on whether the economic feasibility, biomass and technology levels are available to cover the adoption. Decentralized production systems (*e.g.*, local demonstration pyrolysis units or on-farm composting) can enable farmers to utilize local biomass residues for creating valuable soil amendments for their farms thus supporting the development of rural bioeconomy as well as sustainable farming systems.^175^ The multidimensional contributions of carbon-based fertilizers towards sustainable and low carbon agricultures are summarized in Table 3.

**Table 3:** Contribution of carbon-based fertilizers to sustainable development goals and low-carbon agricultural indicators

Category	Indicator/metric	Role of carbon-based fertilizers	Alignment with SDGs	References
Environmental indicators	Soil carbon sequestration	Stabilize long-lived carbon in soils, enhance soil organic carbon pools	SDG 13: climate action	176
	Greenhouse gas mitigation	Reduce N_2_O and CH_4_ emissions *via* controlled nutrient release and adsorption	SDG 13: climate action	177
	Nutrient leaching & runoff reduction	Retain N, P, K in soil through adsorption and slow release	SDG 6: clean water & sanitation	178
	Heavy metal and pollutant immobilization	Adsorb and stabilize toxic elements and organic contaminants in soil	SDG 15: life on land	179
Agronomic benefits	Soil fertility enhancement	Improve CEC, pH buffering, and soil structure	SDG 2: zero hunger	180
	Nutrient use efficiency	Controlled nutrient release synchronizing with crop demand	SDG 2: zero hunger	181
	Plant stress tolerance	Enhance drought, salinity, and heat resilience through rhizosphere modulation	SDG 2 & 13	182
	Microbial and rhizosphere health	Support beneficial microorganisms, PGPR stabilization, biofilm development	SDG 15: life on land	76
Economic outcomes	Input cost reduction	Lower synthetic fertilizer requirement *via* efficient nutrient delivery	SDG 8: decent work & economic growth	183
	Waste valorization	Transform agricultural, municipal, and industrial biomass into fertilizers	SDG 12: responsible consumption & production	184
	Farm productivity & profitability	Improve crop yield and stability under environmental stresses	SDG 2 & 8	185
Policy & sustainability indicators	Climate-precision nutrient management adoption	Integrates low-carbon fertilization, nutrient recycling, and soil carbon management	SDG 13 & 12	186
	Carbon credit potential	Enables farmers to participate in carbon markets *via* biochar application	SDG 13: climate action	187

Reported quantitative outcomes should be interpreted in the context of the specific carbon material, application rate, soil–crop system, experimental duration, and performance metric, because substantial methodological heterogeneity among studies prevents direct comparison or derivation of universal performance ranges.

## 10. Environmental risks, standardization and regulatory challenges

Although the interest in the use of CBFs as sustainable alternatives to conventional fertilizers has been growing, there are still a number of environmental and regulatory barriers that could hinder their widespread application. These obstacles are above all a problem of inconsistencies in the derivation of feedstocks, a lack of coherence of the production methodologies and of long-term ecological analyses, and a lack of harmonized regulatory frameworks. Mitigation against these difficulties is crucial to ensure that the benefits of CBFs provide homogenous agronomic values and do not threaten environmental security.^10^

A major issue associated with CBFs is feedstock variability which can have a significant impact on the physicochemical and agronomic characteristics of the final product. Biomass sources – including agricultural residues, manure, municipal refuse or industrial by-products – vary greatly in their elemental contents, nutrient content and concentrations of contaminants.^170^ Consequently, the nutrient release, stability and environmental behavior of CBFs created from different feedstocks may be heterogeneous. Moreover, some of feedstocks could bring potentially dangerous materials like heavy metals, polycyclic aromatic hydrocarbons (PAHs), and residual organic pollutants which may accumulate into soils if not taken care of.^188^

Another important limitation is the lack of globally harmonized standards related to production, characterization and utilization of carbon-based fertilizers. While a number of countries have introduced guidelines for certain products such as biochar and compost, standardized recommended protocols for estimating properties of *e.g.* carbon stability, nutrient content, contaminant limits and agronomic performance are still lacking.^189^

This lack of standardization makes it difficult to certify products, and to provide assurance of quality and cross study comparison of commercial commodities. Long terms ecological effects are also a little explored area. Although the short-term investigations suggest mostly positive effects of carbon amendments on soil fertility and crop yields, long term consequences of repeated application of CBF are poorly understood. Potential risks include progressive accumulation of contaminants, soil chemical equilibrium change and nutrient cycling change, *etc.* over long periods of time. The use of carbon-based fertilizers could have additional effects on the biodiversity of the soil and on the dynamics of the microbial population.^190,191^

There are still regulatory gaps that challenge the commercialization and uptake of carbon-based fertilizers. In many jurisdictions, such products have an ambiguous legal position in between regulation of soil amendments, fertilizers and substances deriving from waste, which creates uncertainties around classification, safety assessment and market approval of such products.^192^ Regulatory frameworks for use of CBFs in modern agriculture explicit regulatory frameworks, standardized quality criteria and comprehensive environmental risk assessments are therefore indispensable tools to ensure the safe and effective integration of CBFs in modern agricultural systems.^193^

## 11. Future perspectives: towards smart carbon fertilization

Future development of CBFs is expected to take the next step from traditional soil amendments, towards smart and data-driven nutrient solutions with advanced materials science, biotechnology and digital agriculture. Emerging technologies such as artificial intelligence (AI) may speed up the design of fertilizers, predicting the best carbon structure and nutrient loading capabilities and kinetics of release based on the feedstock characteristics and soil conditions. AI-aided modelling could also allow screening of the different biomass conversion pathways at high speeds to engineer carbon matrices with specific physicochemical properties adapted to crops and environments.

One more path that is getting a lot of attention is the ability to integrate CBFs with precision agriculture technologies. Sensor-based soil monitoring, remote sensing systems, and variable rate fertilizer application systems can be combined with stimuli-responsive carbon-based fertilizers to provide nutrient delivery with spatial and temporal precision. Such approaches could be used to drastically improve nutrient use efficiency without causing too much loss to the environment.

Advances in synthetic biology and microbial engineering provide new opportunities to engineer and synthesize carbon- and microbes hybrid fertilizers. Engineered microbial consortia with abilities to fix nitrogen, solubilize P or mitigate stress can be immobilized in a carbon-matrix to produce multifunctional bioactive fertilizers. These systems would allow for nutrients to be delivered in addition to rhizosphere microbiome engineering at the same time. In parallel, digital soil–carbon modelling frameworks are needed for predictions of long-term interactions of carbon fertilizers, soil properties and ecosystem processes. Integrating field data with predictive models will improve our understanding of carbon stability, nutrient dynamics, and climate-mitigation potential in diverse agroecosystems. Significant laboratory and greenhouse data, the best day by day adoption of CBF technologies requires multifaceted field validation and long much time period efficiency analyses. Future research should therefore focus on standardized field research, lifecycle assessments and conduct economic feasibility studies to test the agronomic, environmental and socio-economic effects of smart carbon fertilization systems in real agriculture field conditions.

## 12. Conclusion

Carbon based fertilizer (CBF) is a revolution for achieving sustainable nutrient management between agricultural productivity and environmental care. This review describes the evolution from traditional carbon amendments *i.e.* biochar and compost, to engineered and responsive nutrient-delivery systems based on carbon to increase soil health, regulate nutrient dynamics and climate resilience. By valorizing various biomass feedstocks, such as agricultural residues, manure and organic wastes, CBFs are used to convert waste into functional fertilizers which also facilitates the circular bio-economy principles, the reduction of dependency to conventional synthetic inputs. The physicochemical characteristics of carbon matrices, which include the porosity, surface functional groups and charge dynamics, play a major role in nutrient retention, controlled release, and interactions with soil minerals. CBFs are also operating as rhizosphere engineering platforms by stabilizing the microbial communities in the rhizosphere, boosting nutrient cycling, modulating plant metabolic pathways and mechanisms of stress effects. These mechanisms are found to boost within the plant the nutrient use efficiency, increase in crop growth and resist abiotic stresses such as drought, salinity and heat *etc.*, collectively. There are advances in nano disappears and stimuli despite responsive carbon fertilizers which enhance the precision of nutrient delivery, electron move, and redox investigate mediated nutrient access of soil systems. Environmentally CBFs play a role for the sequestration of carbon, mitigating greenhouse gas emissions, immobilizing pollutants and restoring soil, which is in line with climate smart agricultural goals. Their incorporation into low-carbon farming systems is also assists take policy frameworks for carbon credit and sustainable resource management. Nevertheless, there are still difficulties to overcome including variability of feedstock, lack of standardized production protocol, long-term ecological risks and regulatory gaps. Future research should focus on interdisciplinary approaches including approaches that combine materials science, microbiome engineering, digital agriculture and large-scale field validation approaches to optimize fertilizer performance. Carbon-based fertilizers offer a highly multifunctional platform for sustainable, low-carbon, and climate-resilient agriculture, a critical innovation in changing the soil for increased fertility, environmental impacts reduction, as well as aiding global food and climate security. CBFs offer a highly multifunctional platform for sustainable, low-carbon, and climate-resilient agriculture, a critical innovation in changing the soil for increased fertility, environmental impacts reduction, as well as aiding global food and climate security.

## Author contributions

All authors contributed to the study conception and design. Othman Al-Dossary: writing – original draft preparation and writing – review & editing. Rashid Abbas Khan: writing – original draft preparation, formal analysis, software, and literature synthesis. Tauqeer Ahmed Qadri: writing – original draft preparation, conceptualization, software, visualization, and writing. Abdul Waheed: validation and writing – review & editing. Bader Alsubaie & Wael F. Shehata: resources, validation, and writing – review & editing. Jameel M. Al-Khayri: supervision, project administration, and writing – review & editing. Amjid Khan: conceptualization, supervision, project administration, visualization, software, validation, and writing – review & editing.

## Conflicts of interest

The authors declare that they have no known competing financial interests or personal relationships that could have appeared to influence the work reported in this paper.

## Abbreviations

ALMT: Aluminum-activated malate transporter
CEC: Cation exchange capacity
DOC: Dissolved organic carbon
IoT: Internet of things
MATE: Multidrug and toxic compound extrusion
PGPR: Plant growth-promoting rhizobacteria
SOC: Soil organic carbon

## Data Availability

No primary research results, software or code have been included and no new data were generated or analysed as part of this review.
